# Whole-genome methylation analysis of testicular germ cells from cryptozoospermic men points to recurrent and functionally relevant DNA methylation changes

**DOI:** 10.1186/s13148-021-01144-z

**Published:** 2021-08-21

**Authors:** Sara Di Persio, Elsa Leitão, Marius Wöste, Tobias Tekath, Jann-Frederik Cremers, Martin Dugas, Xiaolin Li, Gerd Meyer zu Hörste, Sabine Kliesch, Sandra Laurentino, Nina Neuhaus, Bernhard Horsthemke

**Affiliations:** 1 Centre of Reproductive Medicine and Andrology, University Hospital of Münster, 48149 Münster, Germany; 2 Institute of Human Genetics, University Hospital Essen, Essen, Germany; 3 Institute of Medical Informatics, University Hospital of Münster, 48149 Münster, Germany; 4 Centre of Reproductive Medicine and Andrology, Department of Clinical and Surgical Andrology, University Hospital of Münster, 48149 Münster, Germany; 5 Department of Neurology, Institute of Translational Neurology, University Hospital of Münster, 48149 Münster, Germany; 6 Institute of Human Genetics, University Hospital Münster, Münster, Germany

**Keywords:** Cryptozoospermia, Testicular germ cells, DNA methylation, Differentially methylated regions, Single cell RNA sequencing, Spermatogenesis, Male infertility

## Abstract

**Background:**

Several studies have reported an association between male infertility and aberrant sperm DNA methylation patterns, in particular in imprinted genes. In a recent investigation based on whole methylome and deep bisulfite sequencing, we have not found any evidence for such an association, but have demonstrated that somatic DNA contamination and genetic variation confound methylation studies in sperm of severely oligozoospermic men. To find out whether testicular germ cells (TGCs) of such patients might carry aberrant DNA methylation, we compared the TGC methylomes of four men with cryptozoospermia (CZ) and four men with obstructive azoospermia, who had normal spermatogenesis and served as controls (CTR).

**Results:**

There was no difference in DNA methylation at the whole genome level or at imprinted regions between CZ and CTR samples. However, using stringent filters to identify group-specific methylation differences, we detected 271 differentially methylated regions (DMRs), 238 of which were hypermethylated in CZ (binominal test, *p* < 2.2 × 10^–16^). The DMRs were enriched for distal regulatory elements (*p* = 1.0 × 10^–6^) and associated with 132 genes, 61 of which are differentially expressed at various stages of spermatogenesis. Almost all of the 67 DMRs associated with the 61 genes (94%) are hypermethylated in CZ (63/67, *p* = 1.107 × 10^–14^). As judged by single-cell RNA sequencing, 13 DMR-associated genes, which are mainly expressed during meiosis and spermiogenesis, show a significantly different pattern of expression in CZ patients. In four of these genes, the promoter is hypermethylated in CZ men, which correlates with a lower expression level in these patients. In the other nine genes, eight of which downregulated in CZ, germ cell-specific enhancers may be affected.

**Conclusions:**

We found that impaired spermatogenesis is associated with DNA methylation changes in testicular germ cells at functionally relevant regions of the genome. We hypothesize that the described DNA methylation changes may reflect or contribute to premature abortion of spermatogenesis and therefore not appear in the mature, motile sperm.

**Supplementary Information:**

The online version contains supplementary material available at 10.1186/s13148-021-01144-z.

## Background

Life-long production of sperm through the process of spermatogenesis is supported by spermatogonia, the most undifferentiated adult germ cell type. The pool of spermatogonia ensures male fertility, as these cells have the potential to self-renew as well as to give rise to differentiating daughter cells. Spermatogonia originate from primordial germ cells (PGCs), which are specified very early during embryonic development. These PGCs undergo erasure of DNA methylation, which allows the establishment of sperm-specific DNA methylation profiles during later stages of gametogenesis [1].

Erasure of DNA methylation takes place in two sequential stages. During the initial stage, a global decrease in methylated cytosines occurs, whereas in the second stage methylation is removed from imprinting control regions and meiotic genes [2–4]. In human male PGCs, the epigenetic ground state of global methylation levels has been found in foetuses between 7 and 11 weeks of age [5–7]. Currently, it is not known when the de novo DNA methylation rise commences in human fetal germ cells, [8] but importantly, this process of de novo global methylation continues until well after birth in primates. [9]

A number of publications have reported that male infertility is associated with aberrant sperm DNA methylation profiles, particularly in imprinted genes [10–15]. However, we found no recurrent epimutations by deep bisulfite sequencing analysis of sperm from patients with severely impaired spermatogenesis (*n* = 93) and controls (*n* = 40), combined with whole genome bisulfite sequencing (WGBS) of selected samples [16]. This study, which is one of the largest in this field, rather revealed that the presence of residual somatic DNA in swim-up purified sperm samples and genetic variation are major confounders of methylation studies in sperm. In line with the recommendations made by Åsenius et al. [17], these two confounders need to be considered in prospective studies to clarify if there is indeed an increased prevalence of aberrant methylation in infertile men.

Only few studies have used whole genome bisulfite sequencing (WGBS) to investigate DNA methylation of human spermatozoa at base pair resolution [16, 18–20]. The main findings of the methylation studies were that there is no significant methylation at non-CpG sites, that there are large regions of low methylation in a manner independent of genomic features such as CpG islands (CGIs) and promoters, and that CGI shores in sperm are more shallow compared to CGI shores in embryonal stem cells (ESCs). WGBS has also been performed on PGCs and advanced germ cells (AGCs) of human embryos highlighting, among other features, the importance of DNA methylation at transposons [5]. With regard to the adult germline, Hammoud et al. [21] have studied murine spermatogonia but genome-wide analysis of DNA methylation in testicular germ cells (TGCs) of adult men and, in particular, its comparison to TGCs from patients with impaired spermatogenesis constitutes a research gap.

Publications of high-quality single-cell RNA sequencing (scRNA-seq) transcriptomes obtained from human testicular tissues with intact spermatogenesis [22–25] have greatly advanced our knowledge with regard to the transcriptional changes associated with human germ cell differentiation. Moreover, comparative analyses of scRNA-seq results from men with intact and severely impaired spermatogenesis have provided insight into the molecular mechanisms associated with failure of germ cell differentiation [25, 26]. In order to assess the presence of aberrant epigenetic patterns in TGCs and the potential association with aberrant transcriptional profiles, we combined the two powerful approaches of WGBS data with scRNA-seq in samples with intact and impaired spermatogenesis.

## Results

### Isolation and characterization of human testicular germ cells

**Fig. 1:**
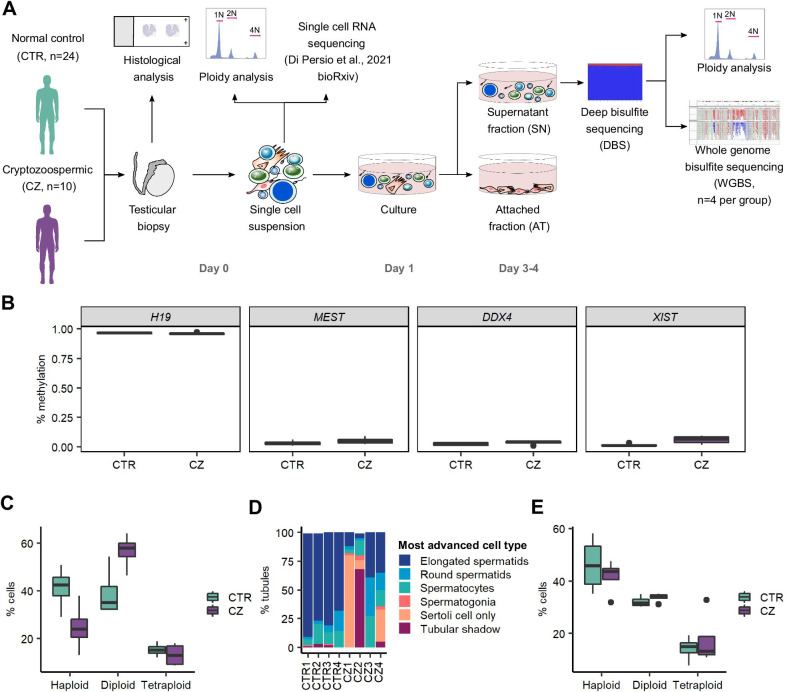
Testicular germ cell samples selection for whole genome bisulfite sequencing. **a** Schematic representation of the experimental design. **b** Box plot showing the methylation values of *H19*, *MEST*, *DDX4* and *XIST* measured using deep bisulfite sequencing (DBS) of the supernatant fraction at day 3–4 of culture for the normal controls (CTR, teal, *n* = 4) and the cryptozoospermic (CZ, purple, *n* = 4) samples. No significant difference was found in the methylation values of the four genes between the two groups. **c** Box plot showing the results of the ploidy analysis of the day 0 single-cell suspension of the normal controls (CTR, teal, *n* = 4) and the cryptozoospermic (CZ, purple, *n* = 4) samples used for whole genome bisulfite sequencing (WGBS). **d** Stacked bar plots showing the percentages of tubules containing germ cells (most advanced germ cell type shown), only Sertoli cells, or tubular shadows in each biopsy from which the samples for WGBS were prepared. **e** Box plot showing the results of the ploidy analysis of the supernatant fraction at day 3–4 of culture for the normal controls (CTR, teal, *n* = 4) and the cryptozoospermic (CZ, purple, *n* = 4) samples. No significant difference was found in the cellular composition of the supernatant fraction between the two groups

To analyse DNA methylation differences between human testicular germ cells (TGCs) in normal and impaired spermatogenesis, we isolated germ cells from patients with obstructive azoospermia (CTR, *n* = 24) and cryptozoospermia (CZ, *n* = 10) (Fig. 1a, Additional file 1: Table S1). By deep bisulfite sequencing (DBS) of *H19*, *MEST*, *DDX4* and *XIST*, we identified 21 CTR (87.5%) and five CZ (50%) samples with pure germ cell fractions, of which we selected four from each group for whole genome bisulfite sequencing (WGBS) (Table 1, Fig. 1a, Additional file 1: Tables S2 and S3, Additional file 2: Fig. S1). These samples were found to have normal methylation values in the four regions, consistent with the absence of somatic DNA, and no significant difference was found between the two groups (Fig. 1b, Additional file 1: Table S3).

**Table 1:** Clinical parameters

Clinical parameters*	CTR1	CTR2	CTR3	CTR4	CZ1	CZ2	CZ3	CZ4
Diagnosis	Obstructive azoospermia				Cryptozoospermia			
Age (Years)	31	33	55	32	39	41	29	23
Testicular volume (ml)	18	25	21	20	7	17	11	16
Total Sperm Count (≥ 39 × 10^6^)	0	0	0	0	< 0.1	< 0.1	< 0.1	< 0.1
FSH (2–10 U/l)	2	3.3	8.2	4.8	8.5	6.5	5.8	3
LH (1–7 U/l)	2.1	2	7.1	3.5	2.5	5.4	6.1	2.6
Total Testosterone (> 12 nmol/l)	16.5	25.1	66	19.9	18.7	13.4	12.7	17.5
Karyotype	46,XY	46,XY	NA	46,XY	46,XY	46,XY	46,XY	NA
Sperm in micro TESE	Yes	Yes	Yes	Yes	Yes	Yes	Yes	Yes
Bergmann-Kliesch score	9	8	8	7	1	0.4	4	3

*FSH *follicle stimulating hormone, *LH *luteinizing hormone, *TESE *testicular sperm extraction. Normal levels of sperm counts and hormones are shown in parentheses

Ploidy analyses of the selected samples showed an enrichment of haploid cells in the CTR samples compared to the CZ samples in the initial single-cell suspension (Fig. 1c). Histological analysis of the testicular biopsies corroborates this result, showing a median of 78.5% and 23.5% of tubules containing elongated spermatids in patients with obstructive azoospermia and cryptozoospermia, respectively (Fig. 1d). Importantly, following 3–4 days of culture, the CTR and the CZ samples had a similar proportion of haploid, diploid, and tetraploid cells in the germ cell fraction (SN), as demonstrated by ploidy analysis (Fig. 1e).

### Whole genome bisulfite sequencing of testicular germ cell DNA from patients with normal and impaired spermatogenesis

Following the coverage recommendations by Ziller et al. [27], we sequenced the eight TGC samples at ~ 14 × coverage each (Additional file 1: Table S4). To further investigate the absence of somatic cell DNA contamination in these TGC samples, we analysed the methylation levels at the 50 known maternally and paternally methylated imprinted control regions (ICR) [28]. As previously shown for uncontaminated sperm samples [16], the eight TGCs showed unmethylated oocyte DMRs and methylated sperm DMRs (Additional file 1: Table S5, Additional file 2: Fig. S2AB), consistent with the absence of somatic DNA contamination.

### Comparison of methylomes from normal TGCs and related cell types

To understand how the methylomes of testicular germ cells compare to the methylomes of related cell types, we first compared the CTR-TGC methylomes with those of normal control sperm samples (*n* = 5) previously generated by our group [16]. Although the same library preparation method and sequencing platform were employed for all methylomes, mean methylation levels were slightly lower in the TGCs than in sperm, both globally and in all genomic features analysed (Additional file 2: Fig. S3AB). Next, we performed a principal component analysis (PCA) using WGBS data from (i) human embryonic stem cell lines (ESCs) (*n* = 2, H1 and H9), (ii) PGC samples collected from 7 to 19 weeks-of-gestation human male embryos (*n* = 8), a developmental time frame at which methylation has been almost completely erased [5], (iii) SSEA4^+^ spermatogonial stem cells (SSCs) isolated from testicular cells by magnetic activated cell sorting (*n* = 2) [29], (iv) CTR-TGC samples from this study (*n* = 4) and (v) sperm samples mentioned above (*n* = 5). The PCA revealed three major clusters: 1. SSCs, TGCs and sperm (upper left hand corner), 2. PGCs (upper right hand corner) and 3. ESCs (lower left hand corner) (Additional file 2: Fig. S3C). Given that the first principal component (PC1) explains 88% of the variability, SSCs, TGCs and sperm have methylation levels much closer to ESCs than to globally demethylated PGCs. Cluster 1 can be further divided into three subclusters corresponding to the different cell types. The SSCs are some distance away from the TGCs and sperm samples, and by investigating the methylation levels of the 34 imprinting control regions (ICRs), which are paternally unmethylated and maternally methylated, we found that the SSC samples from Guo et al. [29], despite having been purified by marker-based sorting, likely contained residual somatic cell DNA (Additional file 2: Fig. S2C). Therefore, we did not consider the SSC datasets for further analyses. The TGCs are slightly separated from sperm, which is in line with their slightly lower global methylation level (see above).

The slightly higher methylation levels of the maternal ICRs in PGCs (Additional file 2: Fig. S2C) are due to the samples collected from 7- and 10-week-old embryos (Additional file 2: Fig. S2D). At this stage, demethylation is probably not yet complete [6], although the presence of somatic cell DNA cannot be excluded.

### Comparison of TGC methylomes from controls and cryptozoospermic men at the global level

**Fig. 2:**
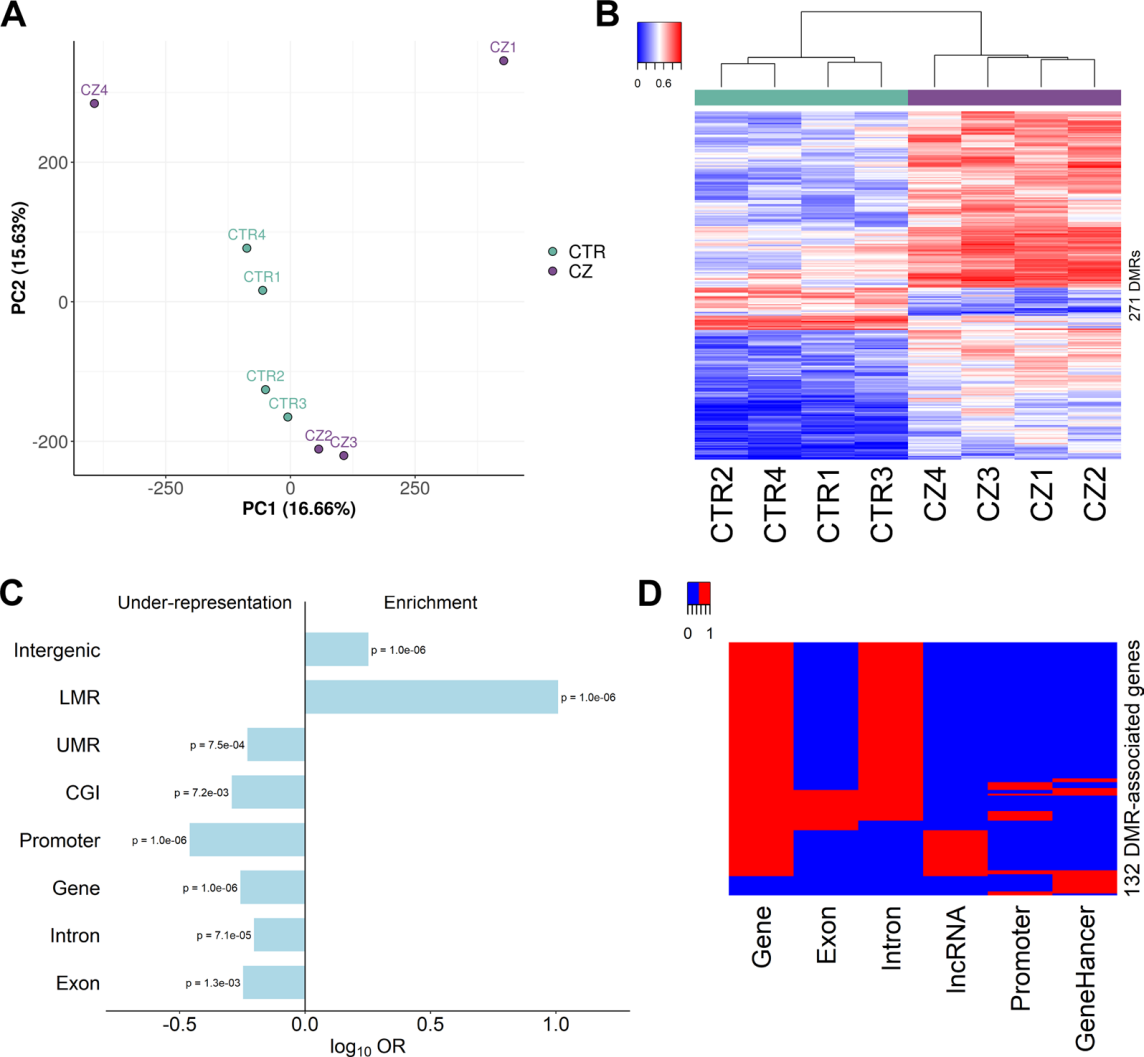
CTR-CZ DMRs are associated with 132 genes. **a** Global comparison of methylomes from control (CTR) and cryptozoospermic (CZ) testicular germ cells. PCA generated for ca. 20 million CpG loci where all samples show methylation values. Only loci with minimum coverage of five in all samples and minimum mapping quality of 10 are considered. CTR testicular germ cell samples in teal, CZ samples in purple. **b** Cluster analysis of the methylation values for the 271 CTR-CZ DMRs in the eight TGC samples. **c** Enrichment/depletion of DMRs for functional genomic regions. LMR, low-methylated region; UMR, unmethylated regions; CGI, CpG islands. **d** DMRs are associated with 132 genes by overlapping genes, promoters and/or “double-elite” enhancers (GeneHancer)

We observed that CTR and CZ methylomes do not differ in their global mean methylation values nor in their overall patterns of methylation in various genomic features (Additional file 1: Table S4, Additional file 2: Fig. S4A, B). Moreover, a PCA of the eight methylomes showed that samples do not cluster according to the diagnosis (Fig. 2a).

For comparing the number and size of proximal and distal regulatory elements in the CTR and CZ genomes, we used MethylSeekR [30] to segment the methylomes into unmethylated regions (UMRs; proximal regulatory regions) and low-methylated regions (LMRs; distal regulatory regions) [31]. We identified on average 36,000 UMRs and 22,000 LMRs per methylome, with CTR and CZ methylomes showing a similar number and total genomic size for each class of regions (Additional file 2: Fig. S4C). Of these, 35,593 UMRs and 14,041 LMRs were found in at least three of the CTR methylomes and were thus considered to be of high confidence (Additional file 2: Tables S6 and S7). Due to the lack of regulatory region annotations for testicular germ cells (unavailability of e.g. histone ChIP-seq data), we used the high-confident UMRs and LMRs as a proxy for putative TGC promoters and enhancers.

### Identification of differentially methylated regions in testicular germ cells from controls and cryptozoospermic men

For identifying group-specific methylation differences between the CTR and CZ samples at the regional level (differentially methylated regions, DMRs), we used three different DMR calling algorithms (metilene, bsmooth, and camel), which typically provide different, but overlapping lists of DMRs. We only considered CpGs that were covered by at least five reads. For metilene, we used *q* > 0.05 as a threshold. For bsmooth and camel, which use t-statistics for identifying differentially methylated CpGs, but do not provide q-values for DMRs, we set a threshold of four CpGs as the minimum DMR length and 0.3 as the minimum methylation difference. Using these filters, we identified 1329 different DMRs (Additional file 2: Fig. S5A, Additional file 1: Table S8). For several of these DMRs, the range of methylation values within each group of samples was very high (Additional file 2: Fig. S5B—left). This was also evident in a cluster analysis of the methylation levels for the 1329 DMRs that showed methylated and unmethylated samples of the same group for some DMRs (Additional file 2: Fig. S5C). In addition, the cluster dendrogram shows a CZ sample clustering together with the CTR group. For each DMR, a high methylation range within a group is likely to result from chance or from differences in genetic background, where a genetic variant determining the methylation state of nearby CpGs can lead to low, medium, or high methylation levels, depending on the presence of one of three genotypes in an individual [32]. To reduce this confounder, we limited the range of methylation values to 0.3 within both groups, thus keeping 271 DMRs for which the two diagnosis groups are clearly separated in the dendrogram (Additional file 2: Fig. S5AB—right, Additional file 1: Table S8). Of these DMRs, 238 are hypermethylated in CZ (binominal test, *p* < 2.2 × 10^–16^).

### Functional annotation of DMRs

For functionally annotating the DMRs, we determined their overlap with common genomic features such as genes and exons as well as with high-confidence UMRs and LMRs (Additional file 1: Table S9, Additional file 2: Fig. S5D). Of the 271 DMRs, 128 (47%) overlap a gene and 65 (24%) overlap a TGC regulatory region as determined by segmentation: 34 overlap a UMR corresponding to a proximal regulatory site and 31 overlap a LMR corresponding to a distal regulatory region. Monte Carlo simulations for DMR enrichment analysis revealed that DMRs are significantly enriched for intergenic regions and LMRs (*p* = 1.0 × 10^–6^) (Fig. 2c). Some DMRs (11%) overlap GeneHancer DoubleElite regulatory elements (Additional file 1: Table S9), which comprise high-confident human enhancers/promoters with high confident association to their gene targets [33].

Based on the intersection of DMRs with genes and/or regulatory elements, we found that 134 DMRs (49%) are associated with 132 different genes (Fig. 2d, Additional file 1: Table S10). Most of the DMR-associated genes are protein-coding (82%), and the vast majority of the DMRs associated with these genes (70%) overlapped an intron. Smaller fractions of the DMR-associated genes arise from DMRs overlapping promoters (11%) and/or for being GeneHancer associated targets (14%) (Fig. 2d, Additional file 1: Table S10).

### Expression of the DMR associated genes during spermatogenesis

**Fig. 3:**
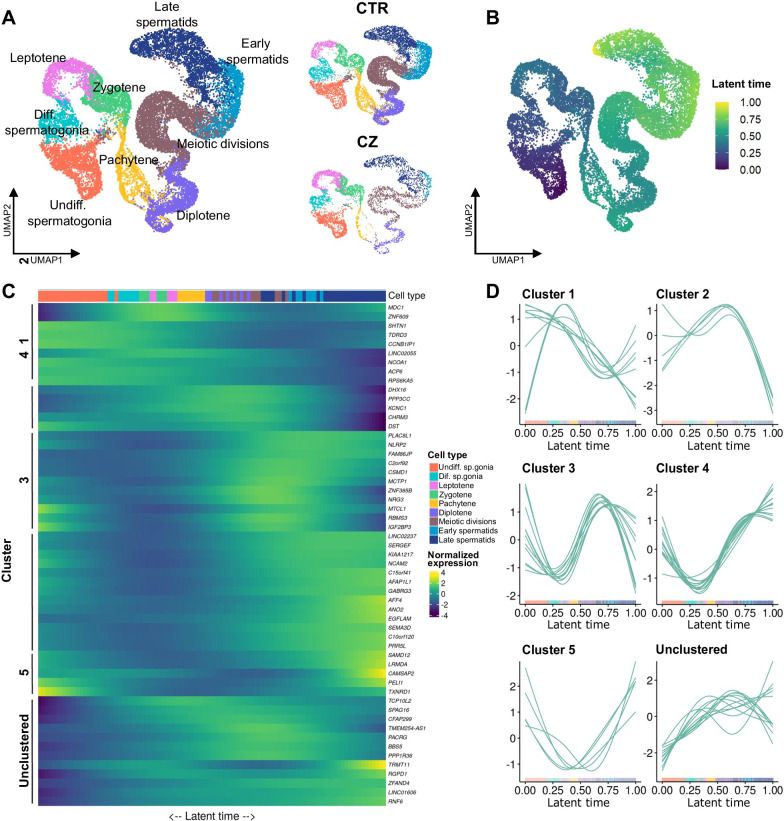
Characterization of the expression of the DMR associated genes in the scRNA-seq datasets. **a** Right panel: uniform manifold approximation and projection (UMAP) plot showing the germ cell subset of the scRNAseq data published in [26]. Left panel: UMAP plots showing the cells obtained from three normal controls (CTR, *n* = 14,098) and from the three cryptozoospermic patient samples (CZ, *n* = 5939). The cells are color coded according to their identity defined by the assignment published in [26]. **b** UMAP plot showing the integrated CTR-CZ germ cell dataset aligned along the latent time. The cells are color-coded according to their progression along the latent time. The undifferentiated spermatogonia cluster was set as starting point of the differentiation process. **c** Heatmap showing the normalized expression of the 55 DMR-associated genes with more than 500 counts in the CTR dataset. The cells are plotted along the latent time with the undifferentiated spermatogonia as starting point on the left side. The clustering analysis identified 5 clusters and left 12 genes unclustered. **d** Line plots showing the normalized expression along the latent time of the 55 DMR-associated genes with high expression grouped according to the belonging cluster in the CTR (teal) dataset

**Fig. 4:**
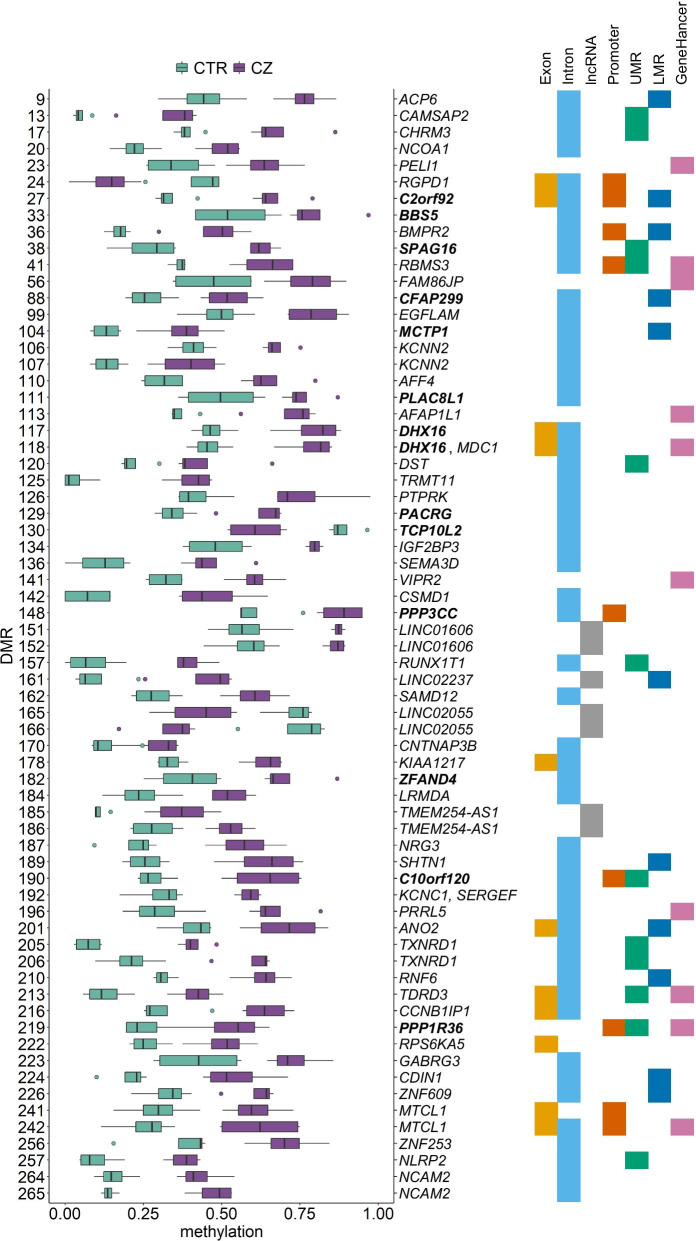
DNA methylation levels and functional characterization of the DMRs associated with genes showing spermatogenesis-regulated expression. Box plots show the distribution of the methylation values for CTR (teal, *n* = 4) and CZ (purple, *n* = 4) (Additional file 1: Table S8). The overlaps of each DMR with specific genomic features are shown: exons (yellow), introns (light-blue), lncRNAs (grey), promoters (orange), UMRs (unmethylated regions, green), LMRs (low-methylated regions, dark-blue) and GeneHancer “double-elite” regulatory regions (pink). The 13 genes in bold were shown to be differentially expressed between CTR and CZ (see Fig. 5)

To ascertain the relevance of the DMR-associated genes, we made use of the available scRNA-seq data obtained by our group from three patients with normal spermatogenesis (CTR) and three patients with cryptozoospermia (CZ) [26]. We subset the germ cells and obtained 14,098 and 5,939 cells from the CTR and CZ groups, respectively (Fig. 3a). We aligned the cells along the latent time, setting the undifferentiated spermatogonia as starting point, and found that it recapitulated the spermatogenic process with the elongated spermatids at the end (Fig. 3b). We then checked the expression of the 132 genes in the normal dataset and found that 55 (41.7%) of them were highly expressed. To identify genes with a similar expression pattern, we performed clustering analysis of the 55 genes. This resulted in the identification of 5 clusters covering 43 of these genes, while 12 genes remained unclustered (Fig. 3c). While the genes in cluster 1 showed their expression peak in spermatogonia, the genes in clusters 2 and 3 had highest expression levels in spermatocytes. Finally, genes in clusters 4 and 5 reached their peak at the spermatid stage (Fig. 3d). We also analysed scRNA-seq data from two other scRNA-seq studies and found that 42 of the 132 genes (32%) are differentially expressed at various stages of spermatogenesis, with 34 reported by Hermann et al. [23] and 40 by Guo et al. [22]. Overall, 61 genes were found in at least one of the three datasets and 29 genes were shared between all three datasets (Additional file 1: Table S10). The 61 spermatogenesis-regulated genes are associated with 67 DMRs, 94% of which are hypermethylated in CZ (63/67, binomial test *p* = 1.107 × 10^–14^) (Fig. 4). The majority of the DMRs are intronic (79%). The other DMRs are located at promoters (13%), exons (16%), and GeneHancer DoubleElite regulatory regions (15%) (Fig. 4), with possible impact of the DMRs on gene expression levels and/or mRNA isoform regulation.

### Differential expression of the DMR associated genes between normal and cryptozoospermic patients

**Fig. 5:**
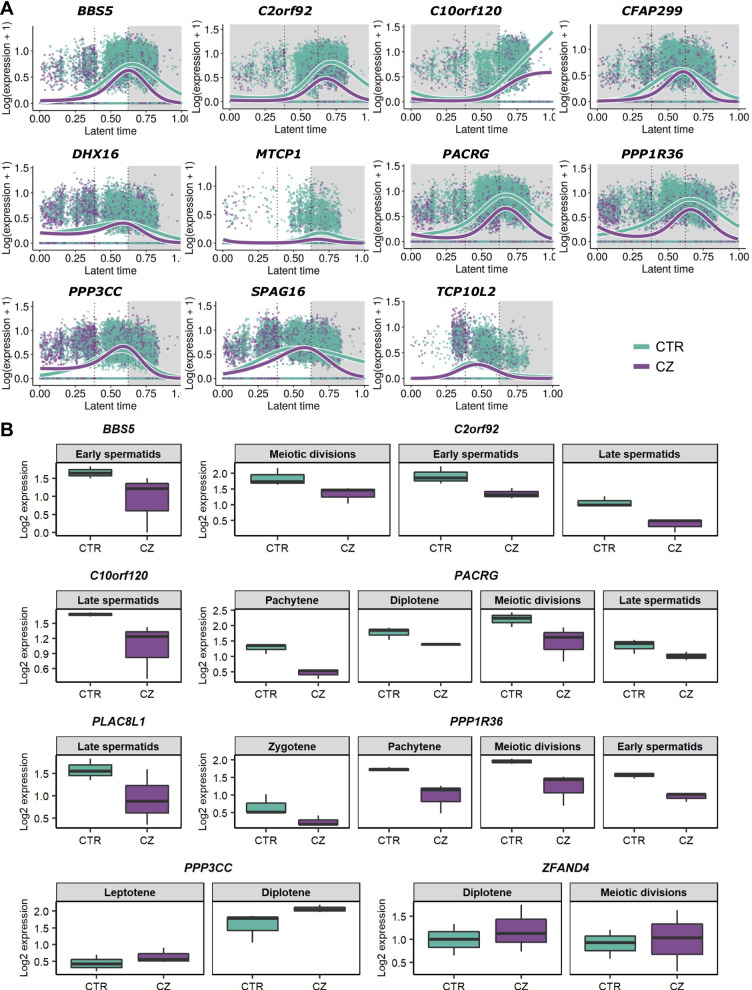
Characterization of the 13 differentially expressed DMR-associated genes between CTR and CZ patients. **a** Line plots showing the expression along the latent time of the 11 DMR associated differentially expressed genes in CTR (teal) and CZ (purple) dataset determined by tradeSeq analysis. The dashed lines mark the knots dividing the three knot groups. The grey areas identify the knot groups in which statistical significance is reached for each gene. Values can be found in Additional file 1: Table S11. **b** Box plots showing the average expression values of the DMR-associated genes that resulted to be differentially expressed using MAST while comparing the CTR (teal, *n* = 3) and CZ scRNA-seq datasets (purple, *n* = 3). Values can be found in Additional file 1: Table S12

To assess whether DNA methylation changes are associated with changes in gene expression levels, we used our recently published differential gene expression analysis of CTR and CZ testicular samples [26]. For this, germ cells were divided into three knot groups (Additional file 2: Fig. S6A) and tradeSeq was used to find trajectory-based differential expression [34]. Of the 132 DMR-associated genes, 11 were differentially expressed (log_2_ fold change > 1 and FDR < 0.01) (Fig. 5a, Additional file 1: Tables S10 and S11). Ten of them were associated with a hypermethylated DMR (Fig. 4, Additional file 2: Fig. S7) and downregulated in CZ (Fig. 5).

To identify in which specific germ cell type the DMR associated genes were differentially expressed, we used the previously published list of differentially expressed genes obtained comparing each CTR germ cell cluster with its respective in the CZ samples [26]. The comparison revealed two additional differentially expressed genes and allowed us to narrow down the differential expression detected by tradeSeq to specific cell type(s) in 6 of the 11 genes (Fig. 5b, Additional file 1: Table S12, Additional file 2: Fig. S6B). Interestingly, with this analysis, no DMR-associated gene was differentially expressed in spermatogonia, but all were differentially expressed during the meiotic stages and/or spermiogenesis. Similarly, the tradeSeq analysis showed all but one gene with expression changes between CTR and CZ testicular samples in the knot group containing cells at later stages of the meiotic divisions and cells undergoing spermiogenesis (Additional file 2: Fig. S6B).

## Discussion

To identify DNA methylation differences associated with impaired spermatogenesis, we generated whole genome methylomes of testicular germ cells (TGCs) of men with cryptozoospermia (CZ) and of men with obstructive azoospermia who had normal spermatogenesis and served as controls (CTR). To the best of our knowledge, there is only one published study focusing on the methylomes of testicular cells from men with reproductive issues and it compared men with obstructive and non-obstructive azoospermia [35]. This study is based on the analysis of testicular biopsies, which comprise both germ cells and somatic cells. The presence of somatic DNA, however, is a major confounder of methylation studies in germ cells [16]. In our study, we separated germ cells from somatic cells by a short-term cell culture and checked the purity of the germ cells by determining the methylation levels of four loci (*MEST*, *H19*, *XIST*, and *DDX4*). In the CZ group, we obtained pure germ cell fractions in only 50% of the cases compared to the 87.5% success rate in the CTR group. This discrepancy is likely due to the increased ratio of somatic cells in the CZ patients and highlights the necessity of a careful isolation of TGC populations especially in patients with impaired spermatogenesis. The analysis of 50 known maternally and paternally methylated imprinted control regions in the WGBS data further confirmed the absence of somatic DNA in the testicular germ cell samples.

First, we compared the methylomes of human testicular germ cells obtained from men with normal spermatogenesis with that of previously published sperm of control individuals [16] confirming that there are no major changes of DNA methylation during spermatogenesis. This was previously described by Guo et al. [29], who showed that the DNA methylation profiles of human spermatogonial stem cells and mature sperm were nearly identical at promoters, putative enhancer sites and imprinted loci. Our comparison of control sperm and testicular germ cell methylomes revealed slightly lower mean methylation levels in testicular germ cells. Although the same library preparation method and sequencing platform were employed in the nine methylomes, the libraries were sequenced in separate runs, which might have introduced a bias. Other possibilities are that the difference is due to a lag in maintenance of DNA methylation by DNMT1 in the newly synthesized daughter DNA strand in dividing TGCs [36], or that the period in culture has an impact on the TGC DNA methylome.

Next, we compared the methylomes of testicular germ cells obtained from controls and cryptozoospermic men. Since unrelated individuals differ in genetic background, which is a major confounder of epigenetic case–control studies in humans, we selected against sequence-based DMRs by applying a range filter for methylation values between samples of the same group. This led to the identification of 271 differentially methylated regions (DMRs). Functional annotation of the 271 DMRs showed that they are enriched for putative distal regulatory regions (LMRs, *p* = 1.0 × 10^–6^) and associated with 132 genes. The analysis of scRNA-seq data on testicular tissues from men with normal spermatogenesis [22, 23, 26] revealed that almost half of them (*n* = 61) are regulated during spermatogenesis and relevant at several stages.

Multiple mechanisms have been suggested on how DNA methylation modulates gene expression. Our analysis has shown that many of the DMRs associated with the 61 spermatogenesis relevant genes overlap known or putative gene regulatory regions. It has long been established that gene promoter methylation is typically related to a down-regulation of gene transcription. We detected nine DMRs overlapping gene promoters, which may be directly linked to differences in gene expression levels between CTR and CZ TGCs or to changes in transcription initiation from an alternative promoter. Furthermore, DNA methylation at enhancers is established by and/or affects the binding of transcription factors (TF), as in the case of CTCF and NRF1 [37]. A few of the DMRs (*n* = 10) coincide with elite enhancers from the GeneHancer DoubleElite track [33] and six of them have been reported as super-enhancers, clusters of transcriptional enhancers driving cell-type-specific gene expression programs, and fundamental to cell identity [38]. Many of the gene-associated DMRs overlap intronic regions and some coincide with putative distal regulatory regions (LMRs), which suggests they might be as-of-yet unknown, possibly germ cell-specific, enhancer regions. Whether DMRs overlapping elite-enhancers, introns, or LMRs actually act as enhancers regulating gene expression in TGCs remains to be determined. DNA methylation has also been shown to regulate splicing of about 22% of alternative exons (reviewed in [39]), and we identified 11 DMRs overlapping exons, which may have an impact on gene isoform regulation.

To address whether some of these DMRs affect gene expression, we performed differential gene expression analysis of scRNA-seq data and found 13 genes showing differences in expression patterns between CTR and CZ TGCs. It is possible that this number is underestimated, since scRNA-seq cannot accurately quantify lowly expressed genes. Furthermore, the variability of isoforms could not be addressed, since a UMI-based scRNA-seq approach was used, which is particularly suited to quantify expression at the gene level, but prevents isoform discrimination when differences are not located in the small sequenced fraction of the transcript [40].

Four of 13 CTR-CZ differentially expressed genes (*C2orf92*, *C10orf120*, *PPP3CC,* and *PPP1R36*) have hypermethylated promoters in cryptozoospermic patients, which correlates with a lower expression levels in these patients. For the remaining nine genes, eight of which are downregulated, expression changes may be driven by methylation at putative germ cell specific enhancers (Table 2). The 13 genes are differentially expressed during the different steps of meiosis and/or spermiogenesis, which suggests a potential correlation between their dysregulation and spermatogenic failure. Indeed, this patient cohort shows a drastic reduction in germ cell number after pachytene stage [26]. Moreover, the great majority of these 13 genes have been shown to have a role in spermatogenesis or to have enriched expression in the testis (Table 2).

**Table 2:** DMRs relevance in CTR-CZ differentially expressed genes

Gene	DMR location	DMR relevance		Methylation change	Expression change	Gene relevance
*BBS5*	Intron		Enhancer?	Hypermethylated	Downregulated in early spermatids	Necessary for the generation of both cilia and flagella [41]
*C2orf92*	Promoter, exon, intron, LMR		Promoter	Hypermethylated	Downregulated in later stages of spermatogenesis	Enriched in the testis [42]
*C10orf120*	Promoter, intron, UMR		Promoter	Hypermethylated	Downregulated in late spermatids	Enriched in the testis [42]
*CFAP299*	Intron, LMR		Enhancer?	Hypermethylated	Downregulated in spermatogenesis	Encodes a cilia- and flagella associated protein recently shown to be involved in mice spermatogenesis [43]
*DHX16*	GeneHancer element, exon, intron		Enhancer	Hypermethylated	Downregulated in later stages of spermatogenesis	Involved in mRNA splicing [44]
*MCTP1*	Intron, LMR		Enhancer?	Hypermethylated	Downregulated in later stages of spermatogenesis	Exclusively found in sperm samples with which pregnancy was achieved [45]
*PACRG*	Intron		Enhancer?	Hypermethylated	Downregulated from pachytene spermatocytes onwards	Role in sperm differentiation and its deletion causes male infertility in mice [46]
*PLAC8L1*	Intron		Enhancer?	Hypermethylated	Downregulated in late spermatids	Understudied gene enriched in the testis [42]
*PPP3CC*	Promoter, intron		Promoter	Hypermethylated	Upregulated in leptotene and diplotene stages	Testis-specific calcineurin isoform that confers midpiece flexibility during the sperm epididymal transit; ablation causes male infertility in mice [47]
*PPP1R36*	Promoter, GeneHancer element, UMR		Promoter	Hypermethylated	Downregulated in zygotene and pachytene spermatocytes, meiotic divisions and early spermatids	Inhibits phosphatase activity of PP1 complexes and has been implicated in enhancing autophagy during spermatogenesis [48]
*SPAG16*	Intron, UMR		Enhancer?	Hypermethylated	Downregulated in later stages of spermatogenesis	Detected in the sperm flagellum axoneme and necessary for its function [49]
*TCP10L2*	Intron		Enhancer?	Hypomethylated	Downregulated in later stages of spermatogenesis	Enriched in the epididymis and testis [42]
*ZFAND4*	Intron		Enhancer?	Hypermethylated	Upregulation in diplotene and meiotic divisions	Enriched in the testis [42]

Methylation and expression changes refer to comparisons of CZ versus CTR. Gene relevance related to testis/fertility

## Conclusions

Based on our findings, we conclude that TGCs from men with impaired spermatogenesis differ from control TGCs in DNA methylation levels at defined genomic regions, many of which appear to be gene-regulatory elements. Imprinting control regions were not affected, suggesting that testicular sperm extraction (TESE) does not further increase the risk of imprinting defects which is associated with intracytoplasmatic sperm injection (ICSI) and other assisted reproduction techniques. We do not know the cause of the aberrant methylation patterns in CZ men, but one possibility is that the observed methylation changes mediate or reflect gene expression changes involving gene-regulatory circuits at the cellular level. The fact that most of the DMRs are hypermethylated in CZ points to a failure of upregulating important genes during spermatogenesis. As we have not observed recurrent epigenetic defects in sperm of a large cohort of infertile men [16], the methylation changes probably contribute to premature abortion of spermatogenesis, and therefore do not appear in mature sperm. As we considered the motile sperm following swim-up procedure, we cannot exclude that aberrantly methylated sperm is present in the discarded semen fraction. Another possibility is that the methylation changes reflect differences in the relative proportion of different types of germ cells, although they were grossly similar by ploidy analysis. The two possibilities are not mutually exclusive, and future improvements in single-cell methylation analysis may resolve this issue. At present, methods for single-cell methylome analysis are not yet efficient enough for case–control studies. Be that as it may, the disruption of cellular events in CZ germ cells (whatever its cause) has allowed us to highlight the importance of DNA methylation in testicular germ cells and to identify gene regulatory regions that undergo DNA methylation changes during spermatogenesis.

## Methods

### Clinical characterization and selection of testicular biopsies

Prior to surgery all patients underwent full phenotyping by physical examination, hormonal analysis (including luteinizing hormone (LH), follicle stimulating hormone (FSH) and testosterone (T)) [16], semen analysis [50] and genetic analyses (karyotype and screening for azoospermia factor (AZF) deletions). Known genetic causes of infertility, acute infections and tumours were exclusion criteria. Control patients were selected from those diagnosed with obstructive azoospermia due to congenital bilateral absence of the vas deferens (CBAVD) or from those undergoing vasectomy reversals. These patients had no sperm in their ejaculate, normal testicular volumes and normal FSH levels (Additional file 1: Table S1). Cryptozoospermic patients had a sperm concentration of less than 0.1 million/ml (cryptozoospermia) in the ejaculate, a reduced testicular volume and in six of the ten cases elevated FSH levels indicating testicular failure. Detailed clinical information about all the patients are available in Additional file 1: Table S1. Following this selection strategy, we obtained biopsies with qualitatively and quantitatively normal spermatogenesis (*n* = 24; controls, CTR), and with idiopathic reduced spermatogenesis (*n* = 10; cryptozoospermic, CZ) between January 2018 and February 2020. For a selection of these samples (*n* = 3 for CTR and CZ each) scRNA-seq was performed and previously published [26].

### Histological analysis of testicular tissues

For routine diagnostic purposes two testicular biopsies per testis were fixed in Bouin’s solution, paraffin embedded and sectioned at 5 µm. Two independent sections per biopsy were subjected to periodic acid-Schiff/hematoxylin (PAS) staining as previously described [26]. The spermatogenic status was evaluated using the Bergmann and Kliesch score [51].

### Short-term culture for purification of testicular germ cells

A differential plating strategy was applied to separate the testicular germ cells from the somatic cells. For this purpose, testicular biopsies were digested into a single-cell suspension using a two-step enzymatic digestion protocol as previously published [52]. Briefly, testicular tissues were minced with sterilized blades into ~ 1 mm^3^ pieces and incubated in MEMα (22561021, Gibco, Thermo Fisher scientific, Waltham, MA USA) with 1 mg/ml collagenase IA (C9891, Sigma-Aldrich, Merck KGaA, Darmstadt, Germany) at 37 °C for 10 min. The digestion was stopped by addition of MEMα supplemented with 10% foetal bovine serum (FBS) (S0615 Merck KGaA, Darmstadt, Germany) and 1% Penicillin/Streptomycin (P/S) (15140–148, Gibco, Thermo Fisher scientific, Waltham, MA USA). After centrifugation the cells were incubated in Hank’s balanced salt solution (14175053, Gibco, Thermo Fisher scientific, Waltham, MA USA) containing 4 mg/ml of trypsin (27250018, Gibco, Thermo Fisher scientific, Waltham, MA USA) and 2.2 mg/ml of DNase I (DN25, Sigma-Aldrich, Merck KGaA, Darmstadt, Germany) at 37 °C for 10 min. Finally, a single-cell suspension was obtained by strong pipetting. The reaction was stopped as outlined above and cells were washed three times with MEMα containing 10% FBS and 1% P/S. The erythrocytes were removed with a three-minute incubation in haemolysis buffer (0.83% NH4Cl solution).The reaction was stopped as outlined above. At the end of the procedure the cells were passed through a 70 µm Sterile CellTrics® filter (Sysmex) and counted using the trypan blue exclusion method. 20,000 cells of the single-cell suspension were stored at −80 °C for ploidy analysis, 12,000 cells were used for scRNA-seq [26] while the rest was plated at a density of < 50.000 cells/cm^2^ onto uncoated cell culture dishes and cultured in MEMα containing 10% FBS and 1% P/S at 35 °C and 5% CO_2_. After 1 day of culture the cells from the supernatant (SN, Germ cells) were separated from the attached cells (AT, Somatic cells) to obtain a pure germ cell fraction [52, 53]. After 3–4 days of culture 20,000 cells of the germ cell fraction were stored at −80 °C for ploidy analysis. The remaining cells were stored at -80 °C for subsequent DNA isolation and DNA methylation analyses.

### Ploidy analysis for analysis of cellular composition

Ploidy analyses of single-cell suspensions obtained immediately after tissue digestion and after 3–4 days of culture were performed to evaluate the cellular composition of each sample. 20,000 cells per sample were processed and analysed as previously published [26].

### DNA isolation and targeted deep bisulfite sequencing for purity screening

To screen the collected germ cell fractions for presence of somatic cell DNA, the following approach was applied. DNA was purified from cultured germ cell fractions using the MasterPure DNA purification kit (MC85200, Epicentre Biotechnologies, Madison, WI, USA) according to the manufacturer’s protocol. DNA concentration was measured using a fluorescence plate reader (FLUOstar Omega, BMG Labtech, Germany). The bisulfite conversion and the deep bisulfite sequencing for *MEST*, *H19* (CTCF6 region)*, XIST* and *DDX4* were performed as previously described [16]. The primer pairs and PCR conditions are described in Additional file 1: Table S2 and were based on previous publications [54–56]. CTR (*n* = 21) and CZ (*n* = 5) samples resulted to have pure germ cell fractions.

### Whole genome bisulfite sequencing

WGBS libraries were prepared from CTR and CZ samples (*n* = 4 each) using 10 ng testicular germ cell DNA supplemented with 1% unmethylated lambda-DNA (Promega, Madison, USA) according to a previously described tagmentation-based method [16], which was based on the protocols described by Wang et al. [57] and Souren et al. [58]. The libraries were sequenced in HiSeq4000 100-bp paired end runs (Illumina, San Diego, USA) using one lane per sample.

### WGBS data analysis

We used wg-blimp v0.9.4 to process WGBS data [59]. In brief, wg-blimp integrates established algorithms for processing WGBS data. These include algorithms for alignment, quality control, DMR calling, methylome segmentation and annotation based on USCS and Ensembl databases [60, 61]. All data were aligned and annotated against human reference hg38. We used R 3.6.0 to import wg-blimp methylation reports and perform PCA analysis on CpG loci where all samples showed at least 5 × coverage.

DMRs were required to cover at least 4 CpG sites with at least 30% methylation difference in the groups compared, minimum mean coverage of 5 × and a maximum *q*-value of 0.05 (where available). We merged DMRs using the GenomicRanges R package [62]. Different filtering strategies based on the ranges of methylation values in CTR and CZ groups were further applied.

DMR overlap with genomic features was compared to simulated DMR distributions to identify potential over- or under-representation of overlap. Specifically, we simulated 1,000,000 sets of DMRs to compare the number of overlaps with genomic features between random and actual DMRs. To create a random set of DMRs we randomly selected 1000 DMRs with the same number of covered CpGs for each of our actual DMRs. From these 1000 DMRs one is selected according to a log-normal distribution of covered nucleotides based on our actual DMR sizes to ensure matching size distributions of simulated and actual DMRs. These simulated sets of DMRs then allow computation of empirical p values and odds ratios for over- and under-representation of overlap.

Putative regulatory regions were identified by segmenting each methylome with MethylSeekR [30], which is part of the wg-blimp pipeline. First, we identified and masked regions of disordered methylation (partially methylated domains, PMDs) and then identified unmethylated regions (UMRs) and low-methylated regions (LMRs). High-confidence UMRs and LMRs were defined as the overlapped regions present in at least three CTR methylomes.

We used the GeneHancer DoubleElite track as a source of highly-ranked human regulatory elements and their inferred target genes, due to their high-likelihood enhancer definition and a strong enhancer-gene association [33]. Repeats refer to elements from RepeatMasker [63].

### ScRNA-seq analysis

The pre-processing, quantification, integration, dimensional reduction, labelling and the MAST differential gene expression analysis were performed as described in the methods in Di Persio et al. [26]. The expression of genes in the scRNA-seq dataset was assessed after subsetting the germ cells out of the CTR and CZ datasets. The latent time was computed on the integrated dataset of all samples, which results in a common latent time trajectory for both sample groups. To reduce noise and increase statistical power of the tradeSeq differential expression test, we focused on strongly expressed genes with a total expression above 500, excluding 77 of the 132 DMR associated genes (58.3%).

### Differential expression analysis along latent time with tradeSeq

We utilized the R package tradeSeq v1.1.18 [64] to perform a differential expression analysis along the determined gene-shared latent time between the spermatogonial cells of the CTR and CZ datasets. The same expression counts from the previous analysis with MAST v1.10.0 [65] were used for this step. For each gene and lineage, negative binomial generalized additive models were fitted between four time points (knots) of the latent time. These knots are equally distributed among the cell density along the trajectory, with the first and last knot representing the minimal and maximal latent time value, respectively. The four knots can be comprised to three knot groups, where the first knot group consists of all cells between knot one and two, etc. To ensure convergence of the generalized additive model fitting process, we increased the maximal number of iterations to 1000. We focused on identifying genes that only show differential expression in a singular knot group. For this, we adopted the stageR v1.6.0 [66] two-staged testing scheme, using a whole-trajectory *patternTest()* for the screening stage and a *earlyDETest()* for each knot group in the confirmation stage. All tests were performed against a log2 fold change of 1, the stageR correction procedure used an overall false discovery rate of 0.01 and a multiple testing correction using the *holm* method [67]. Additionally, the fitted distributions of the normal lineage for the remaining 55 DMR associated genes were clustered to reveal genes with a common expression pattern. The clustering used 100 cluster points and a minimal cluster size of 5, resulting in five distinct clusters as well as a set of unclustered genes.

### Analyses of spermatogenesis-regulated genes

Spermatogenesis-regulated genes as identified by published scRNA-seq datasets of human testicular biopsies were retrieved from the available supplementary information [22, 23, 26]. Genes shared by these datasets and the DMR-associated genes were obtained after all gene names were converted to HGNC approved symbols [68].

### Statistical analyses

Difference between two independent groups was assessed by Mann–Whitney *U* test followed by Bonferroni correction for multiple testing. Binomial tests were used to assess whether two categories are equally likely to occur. Statistical analysis and graphs plotting were performed using R 4.0.0 [69] and appropriate R packages, namely stats v4.0.0 [69], ggplot2 v3.3.0 [70] and gplots v3.0.3 [71].

## Supplementary Information

**Additional file 1**.** Table S1**: Clinical parameters.** Table S2**: Primers for generating amplicons for targeted bisulfite-sequencing.** Table S3**: MEST, H19, XIST and DDX4 methylation values obtained by DBS for the testicular sperm samples.** Table S4**: WGBS statistics. Quality control parameters were based on QualiMap and MethylDackel reports.** Table S5**: WGBS methylation values for the 50 imprinting controls regions.** Table S6**: High-confidence TGC unmethylated regions.** Table S7**: High-confidence TGC low-methylated regions.** Table S8**: WGBS methylation values for the 1329 DMRs.** Table S9**: Annotations for the 271 CTR-CZ DMRs.** Table S10**: DMR-associated genes.** Table S11**: Differentially expressed DMR-associated genes using tradeSeq.** Table S12**: Differentially expressed DMR-associated genes using MAST.

**Additional file 2**.** Fig. S1**: Screening for somatic DNA contamination of the testicular germ cell (TGC) samples. Dotplots representing the mean methylation levels of MEST and H19 (left) and XIST and DDX4 (right) measured by deep bisulfite sequencing in 24 normal controls (CTR, teal) and 10 cryptozoospermic (CZ, purple) testicular germ cell (TGC) samples. ** Fig. S2**: DNA methylation levels in imprinting control regions. A) Methylation levels of the 50 ICRs in the CTR and CZ samples. * Not imprinted according to this data, ** Possible polymorphism. B) Box plots showing the distribution of methylation levels of the 34 oocyte DMRs in the four CTR (teal) and four CZ testicular germ cell samples (purple). C) Comparison of the distributions of the average methylation levels of the 34 oocyte DMRs in the human embryonic stem cells (ESC, * n* = 2), the SSEA+ spermatogonial stem cells from Guo et al. [29] (SSC, * n* = 2), the primordial germ cells isolated from 7–19-week-old embryos datasets from Guo et al. [6] (PGC, * n* = 8) and the CTR and CZ testicular germ cell samples (TGC, * n* = 8, black). D) Comparison of the distribution of the methylation levels of the 34 oocyte DMRs in the eight primordial germ cells samples isolated from 7–19-week-old embryos [6]. Values can be found in Additional file 1: Table S5. Box plots elements are defined as follows: center line: median; box limits: upper and lower quartiles; whiskers: 1.5× interquartile range; points: outliers. ** Fig. S3**: Global comparison of methylomes of control testicular germ cells and control sperm. A) Box plots showing the distribution of global methylation values in control testicular germ cells (CTR, * n* = 4, Additional file 1: Table S4) and sperm normal control samples (SP, * n* = 5, [16]). Statistical analysis showed difference between the two groups (Mann-Whitney U test). Box plots elements are defined as follows: center line: median; box limits: upper and lower quartiles; whiskers: 1.5× interquartile range; points: outliers. B) Violin plots showing the distribution of the mean methylation values for various genomic features in control testicular germ cells (CTR, * n* = 4) and sperm normal control samples (SP, * n* = 5, [16]). Promoters were defined as the 2,000 bp region around TSSs. GeneHancer regions refer to the DoubleElite regulatory elements. Repeats refer to elements from RepeatMasker. C) PCA generated for 1,350,244 CpG loci where all samples show methylation values. Only loci with minimum coverage of five in all samples and minimum mapping quality of 10 are considered. ESC, embryonic stem cells; SSC, spermatogonial stem cells [29]; PGC, primordial germ cells [6][5]; TGC, control testicular germ cells; SP, sperm [16]. ** Fig. S4**: Global comparison of methylomes from control and cryptozoospermic testicular germ cells. A) Box plots showing the distribution of the global methylation values in control testicular germ cells (CTR) and cryptozoospermic testicular germ cells (CZ) (Additional file 1: Table S4). Statistical analysis showed no difference between the two groups (Mann-Whitney U test). Box plots elements are defined as follows: center line: median; box limits: upper and lower quartiles; whiskers: 1.5× interquartile range; points: outliers. B) Violin plots showing the distribution of the mean methylation values for various genomic features in control testicular germ cells (CTR, * n* = 4) and cryptozoospermic testicular germ cells (CZ, * n* = 4). Promoters were defined as the 2000 bp region around TSSs. GeneHancer regions refer to the DoubleElite regulatory elements. Repeats refer to elements from RepeatMasker. C) Distribution of the number (left) and total genomic size (right) of unmethylated (UMR) and low-methylated regions (LMR) obtained by segmenting CTR (teal, * n* = 4) and CZ methylomes (purple,* n* = 4) with MethylSeekR. Statistical analysis showed no difference between the two groups (Mann-Whitney U test). ** Fig. S5**: Differentially methylation regions. A) Flow chart of the discovery of differentially methylated regions (DMRs) between the testicular germ cells from controls and cryptozoospermic men. DMRs were identified with camel, metilene and bsmooth requiring coverage of at least 4 CpGs, with at least 30% difference in methylation, minimum coverage of 5 reads and a maximum q-value of 0.05. Filters on the ranges of methylation values in CTR and CZ groups were further applied. B) Scatter plots showing the relation between the range of methylation values within the CTR and the CZ group for each DMR. DMRs are shown as black dots (included) or white dots (excluded) according to filters on the range of methylation values. Left: no range filters applied. Right: CTR and CZ ranges < 0.3. Numbers of considered DMRs are shown above. C) Cluster analyses of the methylation values of the 1,329 DMRs considered without range filters applied. CTR testicular germ cell samples in teal, CZ samples in purple. Arrows indicate a CZ sample clustering together with the CTR group. D) Number of DMRs from the 271 set that overlap specific functional genomic regions. ** Fig. S6**: scRNA seq analysis. A) UMAP plot showing the integrated CTR-CZ germ cell dataset. The cells are colour-coded according to their knot group identity. Knot group 1 includes cells from undifferentiated spermatogonia to pachytene spermatocytes; Knot group 2 includes cells from pachytene spermatocytes to meiotic divisions; Knot group 3 includes cells from meiotic divisions to late spermatids. B) Schematic representation summarizing the results of the tradeSeq and MAST differential expression analyses. Red colour indicates significant down-regulation of a gene, whereas green indicates significant up-regulation. ** Fig. S7**: IGV browser snapshots of WGBS data from control (CTR) and cryptozoospermic (CZ) testicular germ cells showing CTR-CZ DMRs associated with differentially expressed genes. Each DMR is shown as a red region either spanning the entire width (top panels) or with the surrounding genomic regions (lower panels). Only a subset of reads is shown for each sample. Methylated CpGs are shown in red and unmethylated CpGs in blue

## Data Availability

The WGBS datasets generated in the current study are available in the European Nucleotide Archive (ENA) under the accession number PRJEB39301. Additional WGBS datasets were downloaded from public repositories: sperm (ENA PRJEB34432), ESC (ENA PRJEB39534), SSC (GEO GSE92280) and PGC (GEO GSE63818).

## Abbreviations

AGC: Advanced germ cell
AT: Attached cells fraction
AZF: Azoospermia factor
CBAVD: Congenital bilateral absence of the vas deferens
CGI: CpG island
CTR: Control patient
CZ: Cryptozoospermic patient
DBS: Deep bisulfite sequencing
DMR: Differentially methylated regions
ENA: European Nucleotide Archive
ESC: Embryonic stem cell
FSH: Follicle stimulating hormone
ICR: Imprinting control region
LH: Luteinizing hormone
LMR: Low-methylated region
PAS: Periodic acid-Schiff/hematoxylin
PC: Principal component
PCA: Principal component analysis
PMD: Partially methylated domain
PGC: Primordial germ cell
scRNA-seq: Single cell RNA sequencing
SN: Supernatant fraction of cells
SP: Sperm
SSC: Spermatogonial stem cell
T: Testosterone
TF: Transcription factor
TESE: Testicular sperm extraction
TGC: Testicular germ cell
UMAP: Uniform manifold approximation and projection
UMR: Unmethylated region
WGBS: Whole genome bisulfite sequencing

## References

[1] Seisenberger S, Peat JR, Hore TA, Santos F, Dean W, Reik W (2013). Reprogramming DNA methylation in the mammalian life cycle: building and breaking epigenetic barriers. Phil Trans R Soc B.

[2] Hajkova P, Erhardt S, Lane N, Haaf T, El-Maarri O, Reik W (2002). Epigenetic reprogramming in mouse primordial germ cells. Mech Dev.

[3] Seki Y, Hayashi K, Itoh K, Mizugaki M, Saitou M, Matsui Y (2005). Extensive and orderly reprogramming of genome-wide chromatin modifications associated with specification and early development of germ cells in mice. Dev Biol.

[4] Hill PWS, Leitch HG, Requena CE, Sun Z, Amouroux R, Roman-Trufero M (2018). Epigenetic reprogramming enables the transition from primordial germ cell to gonocyte. Nature.

[5] Gkountela S, Zhang KX, Shafiq TA, Liao W-W, Hargan-Calvopiña J, Chen P-Y (2015). DNA Demethylation dynamics in the human prenatal germline. Cell.

[6] Guo F, Yan L, Guo H, Li L, Hu B, Zhao Y (2015). The transcriptome and DNA methylome landscapes of human primordial germ cells. Cell.

[7] Tang WWC, Dietmann S, Irie N, Leitch HG, Floros VI, Bradshaw CR (2015). A unique gene regulatory network resets the human germline epigenome for development. Cell.

[8] Li L, Li L, Li Q, Liu X, Ma X, Yong J (2021). Dissecting the epigenomic dynamics of human fetal germ cell development at single-cell resolution. Cell Res.

[9] Langenstroth-Röwer D, Gromoll J, Wistuba J, Tröndle I, Laurentino S, Schlatt S (2017). De novo methylation in male germ cells of the common marmoset monkey occurs during postnatal development and is maintained in vitro. Epigenetics.

[10] Kläver R, Tüttelmann F, Bleiziffer A, Haaf T, Kliesch S, Gromoll J (2013). DNA methylation in spermatozoa as a prospective marker in andrology. Andrology.

[11] Kuhtz J, Schneider E, El Hajj N, Zimmermann L, Fust O, Linek B (2014). Epigenetic heterogeneity of developmentally important genes in human sperm: Implications for assisted reproduction outcome. Epigenetics.

[12] Laurentino S, Beygo J, Nordhoff V, Kliesch S, Wistuba J, Borgmann J (2015). Epigenetic germline mosaicism in infertile men. Hum Mol Genet.

[13] Marques CJ, Carvalho F, Sousa M, Barros A (2004). Genomic imprinting in disruptive spermatogenesis. Lancet.

[14] Poplinski A, Tüttelmann F, Kanber D, Horsthemke B, Gromoll J (2010). Idiopathic male infertility is strongly associated with aberrant methylation of *MEST* and *IGF2/H19 ICR1*. Int J Androl.

[15] Urdinguio RG, Bayón GF, Dmitrijeva M, Toraño EG, Bravo C, Fraga MF (2015). Aberrant DNA methylation patterns of spermatozoa in men with unexplained infertility. Hum Reprod.

[16] Leitão E, Di Persio S, Laurentino S, Wöste M, Dugas M, Kliesch S (2020). The sperm epigenome does not display recurrent epimutations in patients with severely impaired spermatogenesis. Clin Epigenet.

[17] Åsenius F, Danson AF, Marzi SJ (2020). DNA methylation in human sperm: a systematic review. Hum Reprod Update.

[18] Guo H, Zhu P, Yan L, Li R, Hu B, Lian Y (2014). The DNA methylation landscape of human early embryos. Nature.

[19] Hammoud SS, Low DHP, Yi C, Carrell DT, Guccione E, Cairns BR (2014). Chromatin and transcription transitions of mammalian adult germline stem cells and spermatogenesis. Cell Stem Cell.

[20] Molaro A, Hodges E, Fang F, Song Q, McCombie WR, Hannon GJ (2011). Sperm methylation profiles reveal features of epigenetic inheritance and evolution in primates. Cell.

[21] Hammoud SS, Low DHP, Yi C, Lee CL, Oatley JM, Payne CJ (2015). Transcription and imprinting dynamics in developing postnatal male germline stem cells. Genes Dev.

[22] Guo J, Grow EJ, Mlcochova H, Maher GJ, Lindskog C, Nie X (2018). The adult human testis transcriptional cell atlas. Cell Res.

[23] Hermann BP, Cheng K, Singh A, Roa-De La Cruz L, Mutoji KN, Chen I-C, et al. The Mammalian spermatogenesis single-cell transcriptome, from spermatogonial stem cells to spermatids. Cell Rep. 2018;25:1650–67. 10.1016/j.celrep.2018.10.026 PMC6384825 30404016

[24] Sohni A, Tan K, Song H-W, Burow D, de Rooij DG, Laurent L (2019). The neonatal and adult human testis defined at the single-cell level. Cell Rep.

[25] Wang M, Liu X, Chang G, Chen Y, An G, Yan L (2018). Single-cell RNA sequencing analysis reveals sequential cell fate transition during human spermatogenesis. Cell Stem Cell.

[26] Di Persio S, Tekath T, Siebert-Kuss LM, Cremers J-F, Wistuba J, Li X, et al. EGR4-dependent decrease of UTF1 is associated with failure to reserve spermatogonial stem cells in infertile men [Internet]. Mol Biol; 2021. Available from: http://biorxiv.org/lookup/doi/10.1101/2021.02.02.429371

[27] Ziller MJ, Hansen KD, Meissner A, Aryee MJ (2015). Coverage recommendations for methylation analysis by whole-genome bisulfite sequencing. Nat Methods.

[28] Monk D, Morales J, den Dunnen JT, Russo S, Court F, Prawitt D (2018). Recommendations for a nomenclature system for reporting methylation aberrations in imprinted domains. Epigenetics.

[29] Guo J, Grow EJ, Yi C, Mlcochova H, Maher GJ, Lindskog C (2017). Chromatin and single-cell RNA-seq profiling reveal dynamic signaling and metabolic transitions during human spermatogonial stem cell development. Cell Stem Cell.

[30] Burger L, Gaidatzis D, Schübeler D, Stadler MB (2013). Identification of active regulatory regions from DNA methylation data. Nucleic Acids Res.

[31] Stadler MB, Murr R, Burger L, Ivanek R, Lienert F, Schöler A (2011). DNA-binding factors shape the mouse methylome at distal regulatory regions. Nature.

[32] Schröder C, Leitão E, Wallner S, Schmitz G, Klein-Hitpass L, Sinha A (2017). Regions of common inter-individual DNA methylation differences in human monocytes: genetic basis and potential function. Epigenetics Chromatin.

[33] Fishilevich S, Nudel R, Rappaport N, Hadar R, Plaschkes I, Iny Stein T, et al. GeneHancer: genome-wide integration of enhancers and target genes in GeneCards. Database [Internet]. 2017 [cited 2021 Jul 20];2017. Available from: https://academic.oup.com/database/article/doi/10.1093/database/bax028/3737828 10.1093/database/bax028 PMC5467550 28605766

[34] Van den Berge K, Roux de Bézieux H, Street K, Saelens W, Cannoodt R, Saeys Y, et al. Trajectory-based differential expression analysis for single-cell sequencing data. Nat Commun. 2020;11:1201. 10.1038/s41467-020-14766-3 PMC7058077 32139671

[35] Wu X, Luo C, Hu L, Chen X, Chen Y, Fan J (2020). Unraveling epigenomic abnormality in azoospermic human males by WGBS, RNA-Seq, and transcriptome profiling analyses. J Assist Reprod Genet.

[36] Charlton J, Downing TL, Smith ZD, Gu H, Clement K, Pop R (2018). Global delay in nascent strand DNA methylation. Nat Struct Mol Biol.

[37] Zhu H, Wang G, Qian J (2016). Transcription factors as readers and effectors of DNA methylation. Nat Rev Genet.

[38] Hnisz D, Abraham BJ, Lee TI, Lau A, Saint-André V, Sigova AA (2013). Super-enhancers in the control of cell identity and disease. Cell.

[39] Lev Maor G, Yearim A, Ast G (2015). The alternative role of DNA methylation in splicing regulation. Trends Genet.

[40] Arzalluz-Luque Á, Conesa A (2018). Single-cell RNAseq for the study of isoforms—how is that possible?. Genome Biol.

[41] Li JB, Gerdes JM, Haycraft CJ, Fan Y, Teslovich TM, May-Simera H (2004). Comparative genomics identifies a Flagellar and basal body proteome that includes the BBS5 human disease gene. Cell.

[42] Uhlen M, Fagerberg L, Hallstrom BM, Lindskog C, Oksvold P, Mardinoglu A (2015). Tissue-based map of the human proteome. Science.

[43] Li H, Dai Y, Luo Z, Nie D (2019). Cloning of a new testis-enriched gene C4orf22 and its role in cell cycle and apoptosis in mouse spermatogenic cells. Mol Biol Rep.

[44] Gencheva M, Kato M, Newo ANS, Lin R-J (2010). Contribution of DEAH-box protein DHX16 in human pre-mRNA splicing. Biochem J.

[45] García-Herrero S, Meseguer M, Martínez-Conejero JA, Remohí J, Pellicer A, Garrido N (2010). The transcriptome of spermatozoa used in homologous intrauterine insemination varies considerably between samples that achieve pregnancy and those that do not. Fertil Steril.

[46] Lorenzetti D, Bishop CE, Justice MJ (2004). Deletion of the Parkin coregulated gene causes male sterility in the quakingviable mouse mutant. Proc Natl Acad Sci.

[47] Miyata H, Satouh Y, Mashiko D, Muto M, Nozawa K, Shiba K (2015). Sperm calcineurin inhibition prevents mouse fertility with implications for male contraceptive. Science.

[48] Zhang Q, Gao M, Zhang Y, Song Y, Cheng H, Zhou R (2016). The germline-enriched Ppp1r36 promotes autophagy. Sci Rep.

[49] Zhang Z, Zariwala MA, Mahadevan MM, Caballero-Campo P, Shen X, Escudier E (2007). A heterozygous mutation disrupting the SPAG16 Gene results in biochemical instability of central apparatus components of the human sperm axoneme1. Biol Reprod.

[50] World Health Organization. World Health Organization.: WHO laboratory manual for the examination and processing of human semen. 5th edn. Geneva; 2010.

[51] Bergmann and Kliesch. Testicular Biopsy and Histology. In: Andrology-Us Edited by Nieschlag E, Behre HM, Nieschlag S; 2010: 155–167. Berlin, Heidelberg: Springer; 2010. p. 155–67.

[52] Neuhaus N, Yoon J, Terwort N, Kliesch S, Seggewiss J, Huge A, et al. Single-cell gene expression analysis reveals diversity among human spermatogonia. Mol Hum Reprod. 2017;molehr;gaw079v2. 10.1093/molehr/gaw079 28093458

[53] Kossack N, Terwort N, Wistuba J, Ehmcke J, Schlatt S, Schöler H (2013). A combined approach facilitates the reliable detection of human spermatogonia in vitro. Hum Reprod.

[54] Beygo J, Citro V, Sparago A, De Crescenzo A, Cerrato F, Heitmann M (2013). The molecular function and clinical phenotype of partial deletions of the IGF2/H19 imprinting control region depends on the spatial arrangement of the remaining CTCF-binding sites. Hum Mol Genet.

[55] Laurentino S, Heckmann L, Di Persio S, Li X, Meyer zu Hörste G, Wistuba J, et al. High-resolution analysis of germ cells from men with sex chromosomal aneuploidies reveals normal transcriptome but impaired imprinting. Clin Epigenet. 2019;11:127. 10.1186/s13148-019-0720-3 PMC6714305 31462300

[56] Rahmann S, Beygo J, Kanber D, Martin M, Horsthemke B, Buiting K. Amplikyzer: Automated methylation analysis of amplicons from bisulfite flowgram sequencing [Internet]. PeerJ PrePrints; 2013. Available from: https://peerj.com/preprints/122v2

[57] Wang Q, Gu L, Adey A, Radlwimmer B, Wang W, Hovestadt V (2013). Tagmentation-based whole-genome bisulfite sequencing. Nat Protoc.

[58] Souren NY, Gerdes LA, Lutsik P, Gasparoni G, Beltrán E, Salhab A (2019). DNA methylation signatures of monozygotic twins clinically discordant for multiple sclerosis. Nat Commun.

[59] Wöste M, Leitão E, Laurentino S, Horsthemke B, Rahmann S, Schröder C (2020). wg-blimp: an end-to-end analysis pipeline for whole genome bisulfite sequencing data. BMC Bioinform.

[60] Haeussler M, Zweig AS, Tyner C, Speir ML, Rosenbloom KR, Raney BJ (2019). The UCSC genome browser database: 2019 update. Nucleic Acids Res.

[61] Yates AD, Achuthan P, Akanni W, Allen J, Allen J, Alvarez-Jarreta J, et al. Ensembl 2020. Nucleic Acids Res. 2019;gkz966. 10.1093/nar/gkz966 PMC7145704 31691826

[62] Lawrence M, Huber W, Pagès H, Aboyoun P, Carlson M, Gentleman R, et al. Software for computing and annotating genomic ranges. Prlic A, editor. PLoS Comput Biol. 2013;9:e1003118. 10.1371/journal.pcbi.1003118 PMC3738458 23950696

[63] RepeatMasker Open-3.0 [Internet]. Available from: http://www.repeatmasker.org

[64] Van den Berge K, Roux de Bezieux H. tradeSeq: trajectory-based differential expression analysis for sequencing data. 2020. 10.1038/s41467-020-14766-3 PMC7058077 32139671

[65] Finak G, McDavid A, Yajima M, Deng J, Gersuk V, Shalek AK (2015). MAST: a flexible statistical framework for assessing transcriptional changes and characterizing heterogeneity in single-cell RNA sequencing data. Genome Biol.

[66] Van den Berge K, Soneson C, Robinson MD, Clement L (2017). stageR: a general stage-wise method for controlling the gene-level false discovery rate in differential expression and differential transcript usage. Genome Biol.

[67] Holm S. A Simple Sequentially Rejective Multiple Test Procedure. Scandinavian Journal of Statistics. [Board of the Foundation of the Scandinavian Journal of Statistics, Wiley]; 1979;6:65–70.

[68] Braschi B, Denny P, Gray K, Jones T, Seal R, Tweedie S, et al. Genenames.org: the HGNC and VGNC resources in 2019. Nucleic Acids Research. 2019;47:D786–92. 10.1093/nar/gky930 PMC6324057 30304474

[69] R: A language and environment for statistical computing [Internet]. Available from: https://www.R-project.org/

[70] Villanueva RAM, Chen ZJ. ggplot2: Elegant Graphics for Data Analysis, 2nd edition. Meas-Interdiscip Res. 2019th ed. 17(3); 2019. p. 160–7.

[71] gplots: Various R Programming Tools for Plotting Data [Internet]. Available from: https://cran.r-project.org/web/packages/gplots/index.html

