# Supplementary material for: Whole-genome methylation analysis of testicular germ cells from cryptozoospermic men points to recurrent and functionally relevant DNA methylation changes
Source: Clin Epigenetics. 2021 Aug 21;13:160. doi: 10.1186/s13148-021-01144-z (PMC8379757; doi:10.1186/s13148-021-01144-z)
Supplement: Supplementary file 2 — Additional file 2. Fig. S1: Screening for somatic DNA contamination of the testicular germ cell (TGC) samples. Dotplots representing the mean methylation levels of MEST and H19 (left) and XIST and DDX4 (right) measured by deep bisulfite sequencing in 24 normal controls (CTR, teal) and 10 cryptozoospermic (CZ, purple) testicular germ cell (TGC) samples. Fig. S2: DNA methylation levels in imprinting control regions. A) Methylation levels of the 50 ICRs in the CTR and CZ samples. * Not imprinted according to this data, ** Possible polymorphism. B) Box plots showing the distribution of methylation levels of the 34 oocyte DMRs in the four CTR (teal) and four CZ testicular germ cell samples (purple). C) Comparison of the distributions of the average methylation levels of the 34 oocyte DMRs in the human embryonic stem cells (ESC, n = 2), the SSEA+ spermatogonial stem cells from Guo et al. [29] (SSC, n = 2), the primordial germ cells isolated from 7–19-week-old embryos datasets from Guo et al. [6] (PGC, n = 8) and the CTR and CZ testicular germ cell samples (TGC, n = 8, black). D) Comparison of the distribution of the methylation levels of the 34 oocyte DMRs in the eight primordial germ cells samples isolated from 7–19-week-old embryos [6]. Values can be found in Additional file 1: Table S5. Box plots elements are defined as follows: center line: median; box limits: upper and lower quartiles; whiskers: 1.5× interquartile range; points: outliers. Fig. S3: Global comparison of methylomes of control testicular germ cells and control sperm. A) Box plots showing the distribution of global methylation values in control testicular germ cells (CTR, n = 4, Additional file 1: Table S4) and sperm normal control samples (SP, n = 5, [16]). Statistical analysis showed difference between the two groups (Mann-Whitney U test). Box plots elements are defined as follows: center line: median; box limits: upper and lower quartiles; whiskers: 1.5× interquartile range; points: outliers. B) Violin [file 13148_2021_1144_MOESM2_ESM.pdf]

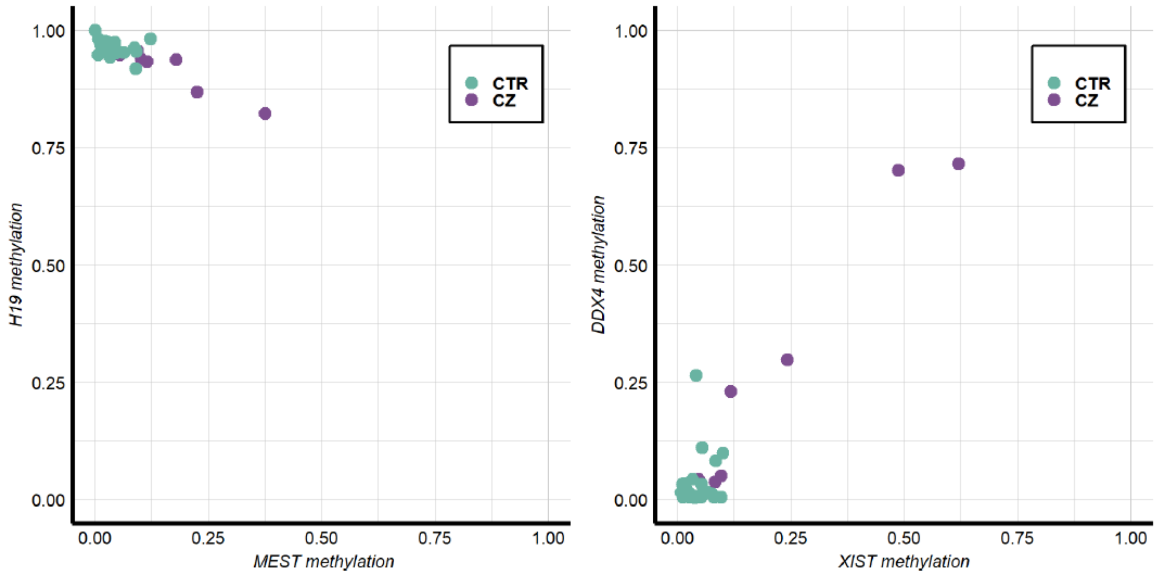

Fig. S1: Screening for somatic DNA contamination of the testicular germ cell (TGC) samples. Dotplots representing the mean methylation levels of *MEST* and *H19* (left) and *XIST* and *DDX4* (right) measured by deep bisulfite sequencing in 24 normal controls (CTR, teal) and 10 cryptozoospermic (CZ, purple) testicular germ cell (TGC) samples.

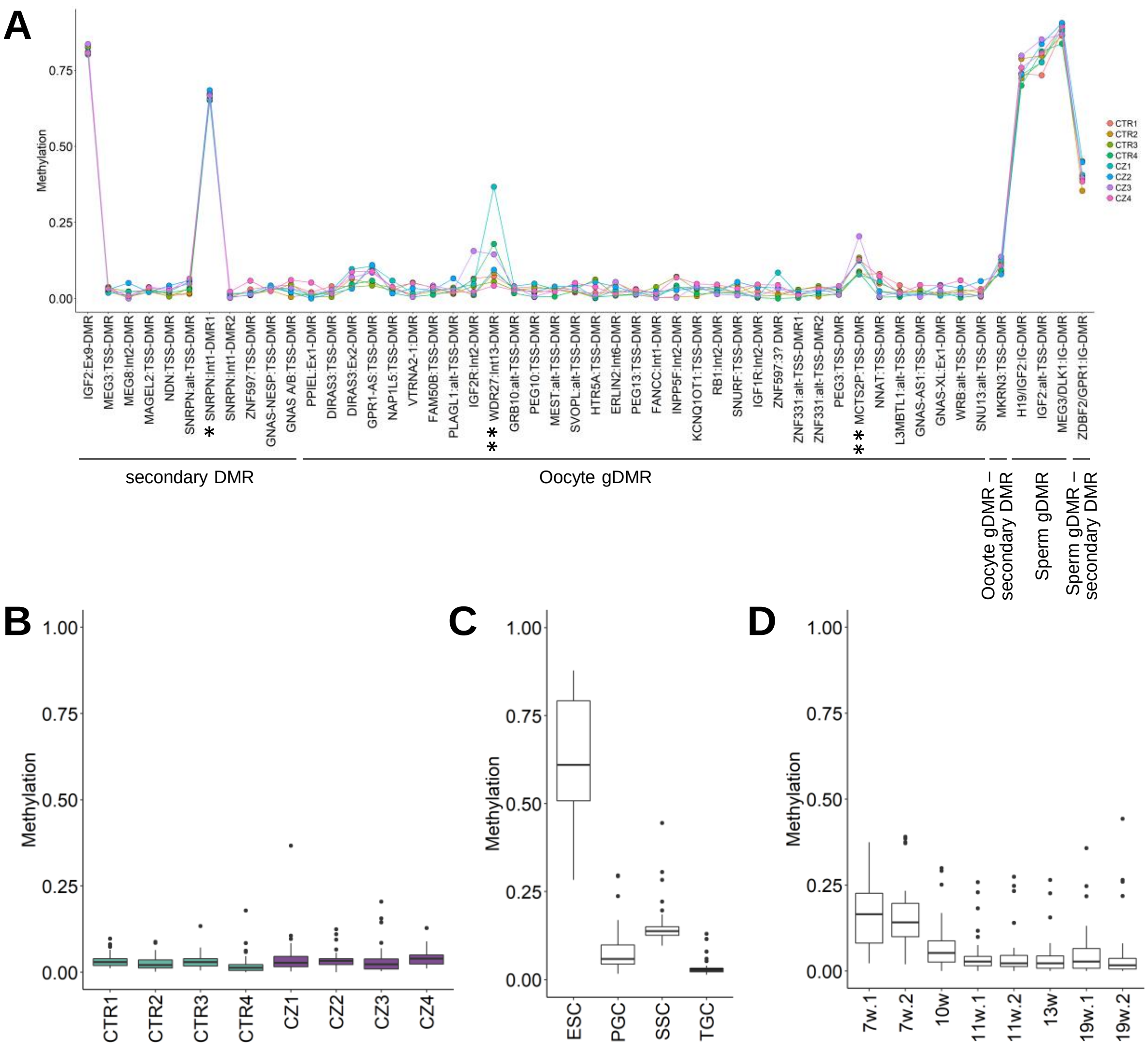

Fig. S2: DNA methylation levels in imprinting control regions. A) Methylation levels of the 50 ICRs in the CTR and CZ samples. * Not imprinted according to this data, ** Possible polymorphism. B) Box plots showing the distribution of methylation levels of the 34 oocyte DMRs in the four CTR (teal) and four CZ testicular germ cell samples (purple). C) Comparison of the distributions of the average methylation levels of the 34 oocyte DMRs in the human embryonic stem cells (ESC, n=2), the SSEA+ spermatogonial stem cells from Guo et al. [29] (SSC, n=2), the primordial germ cells isolated from 7-19 week-old embryos datasets from Guo et al. [5] (PGC, n=8) and the CTR and CZ testicular germ cell samples (TGC, n=8, black). D) Comparison of the distribution of the methylation levels of the 34 oocyte DMRs in the eight primordial germ cells samples isolated from 7-19 week-old embryos [5]. Values can be found in Additional file 1: Table S5. Box plots elements are defined as follows: center line: median; box limits: upper and lower quartiles; whiskers: 1.5× interquartile range; points: outliers.

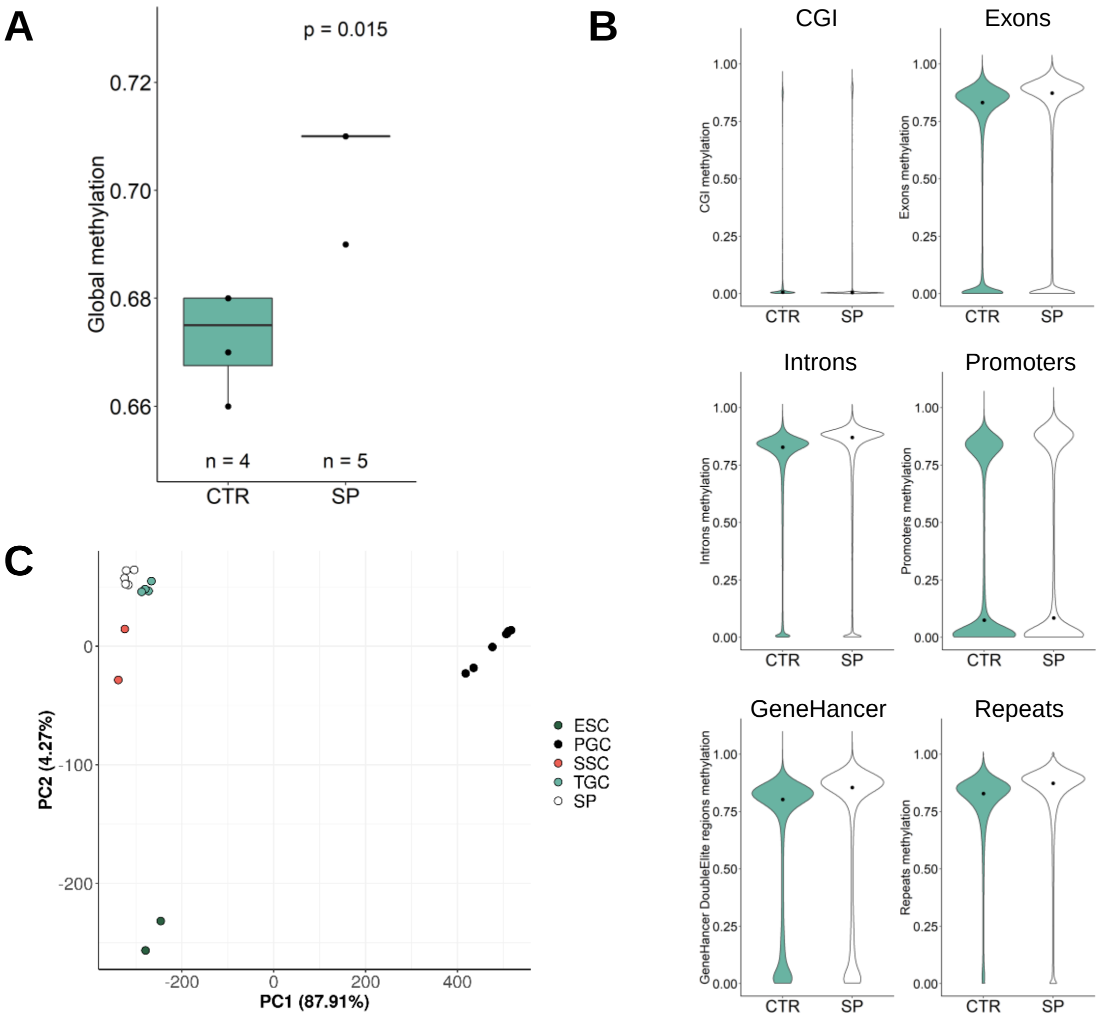

Fig. S3: Global comparison of methylomes of control testicular germ cells and control sperm. A) Box plots showing the distribution of global methylation values in control testicular germ cells (CTR, n=4, Additional file 1: Table S4) and sperm normal control samples (SP, n=5, [14]). Statistical analysis showed difference between the two groups (Mann-Whitney U test). Box plots elements are defined as follows: center line: median; box limits: upper and lower quartiles; whiskers: 1.5× interquartile range; points: outliers. B) Violin plots showing the distribution of the mean methylation values for various genomic features in control testicular germ cells (CTR, n=4) and sperm normal control samples (SP, n=5, [14]). Promoters were defined as the 2,000 bp region around TSSs. GeneHancer regions refer to the DoubleElite regulatory elements. Repeats refer to elements from RepeatMasker. C) PCA generated for 1,350,244 CpG loci where all samples show methylation values. Only loci with minimum coverage of five in all samples and minimum mapping quality of 10 are considered. ESC, embryonic stem cells; SSC, spermatogonial stem cells [29]; PGC, primordial germ cells [5]; TGC, control testicular germ cells; SP, sperm [14].

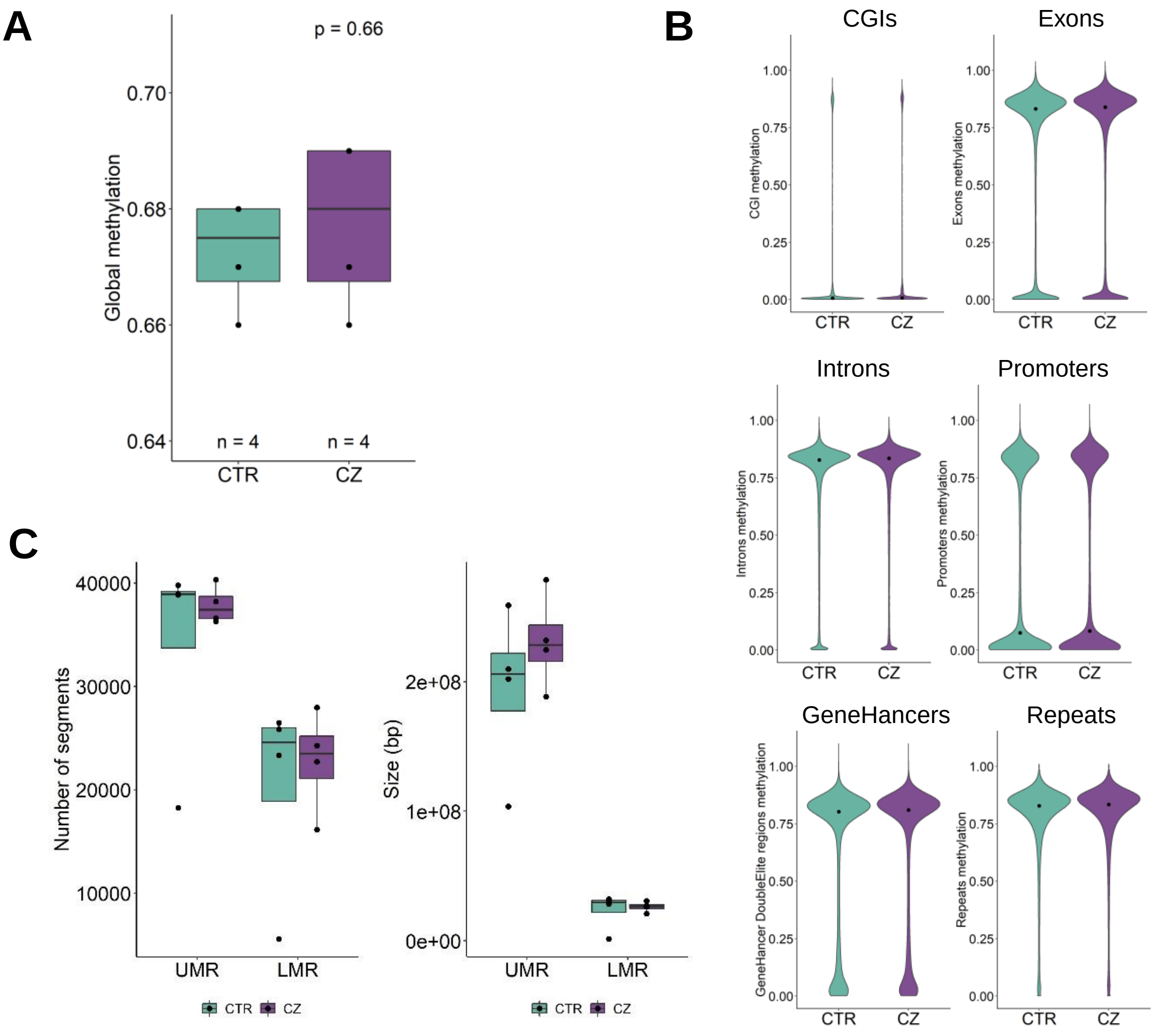

Fig. S4: Global comparison of methylomes from control and cryptozoospermic testicular germ cells. A) Box plots showing the distribution of the global methylation values in control testicular germ cells (CTR) and cryptozoospermic testicular germ cells (CZ) (Additional file 1: Table S4). Statistical analysis showed no difference between the two groups (Mann-Whitney U test). Box plots elements are defined as follows: center line: median; box limits: upper and lower quartiles; whiskers: 1.5× interquartile range; points: outliers. B) Violin plots showing the distribution of the mean methylation values for various genomic features in control testicular germ cells (CTR, n=4) and cryptozoospermic testicular germ cells (CZ, n=4). Promoters were defined as the 2,000 bp region around TSSs. GeneHancer regions refer to the DoubleElite regulatory elements. Repeats refer to elements from RepeatMasker. C) Distribution of the number (left) and total genomic size (right) of unmethylated (UMR) and low-methylated regions (LMR) obtained by segmenting CTR (teal, n=4) and CZ methylomes (purple, n=4) with MethylSeekR. Statistical analysis showed no difference between the two groups (Mann-Whitney U test).

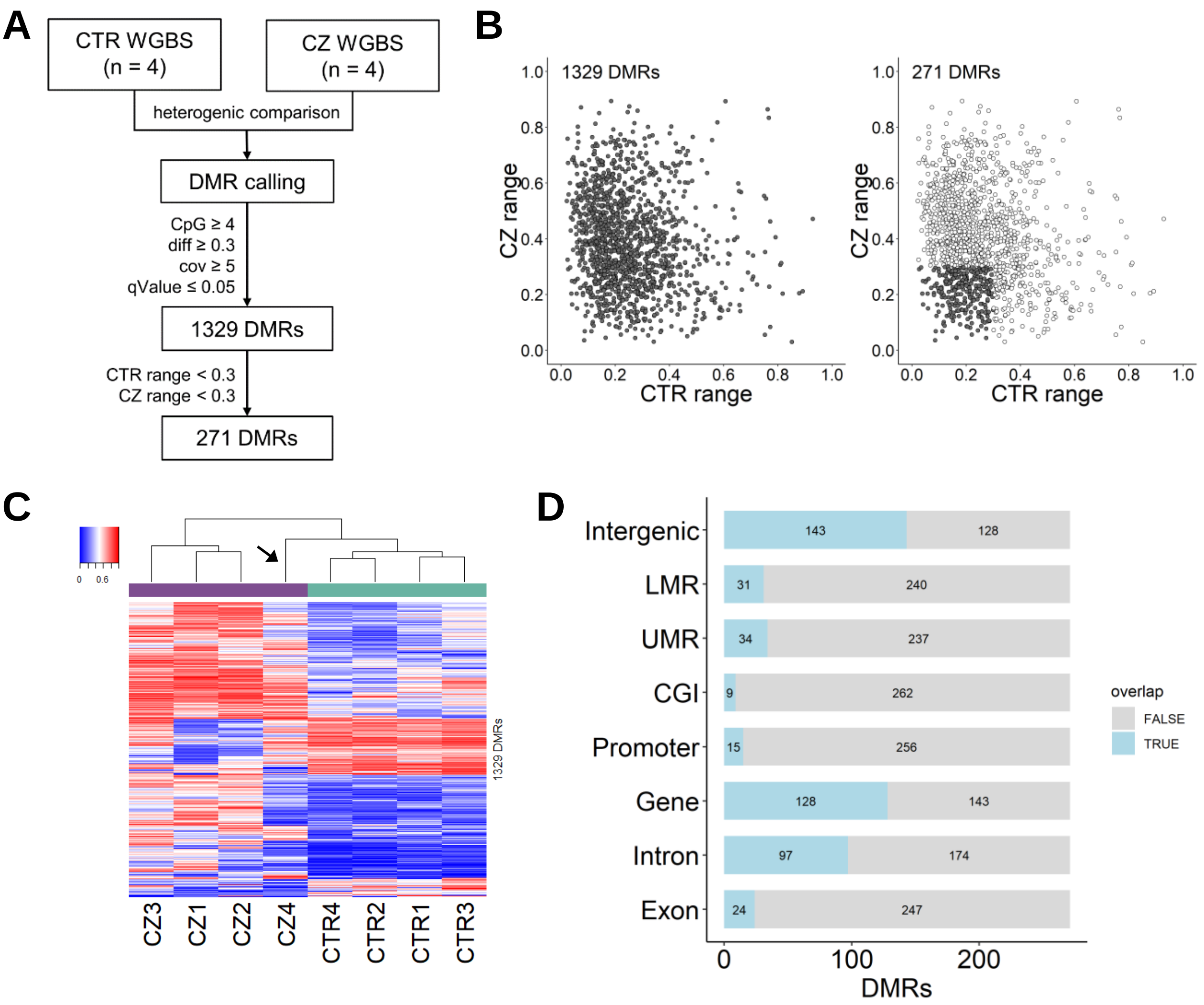

Fig. S5: Differentially methylation regions. A) Flow chart of the discovery of differentially methylated regions (DMRs) between the testicular germ cells from controls and cryptozoospermic men. DMRs were identified with camel, metilene and bsmooth requiring coverage of at least 4 CpGs, with at least 30% difference in methylation, minimum coverage of 5 reads and a maximum q-value of 0.05. Filters on the ranges of methylation values in CTR and CZ groups were further applied. B) Scatter plots showing the relation between the range of methylation values within the CTR and the CZ group for each DMR. DMRs are shown as black dots (included) or white dots (excluded) according to filters on the range of methylation values. Left: no range filters applied. Right: CTR and CZ ranges < 0.3. Numbers of considered DMRs are shown above. C) Cluster analyses of the methylation values of the 1,329 DMRs considered without range filters applied. CTR testicular germ cell samples in teal, CZ samples in purple. Arrows indicate a CZ sample clustering together with the CTR group. D) Number of DMRs from the 271 set that overlap specific functional genomic regions.

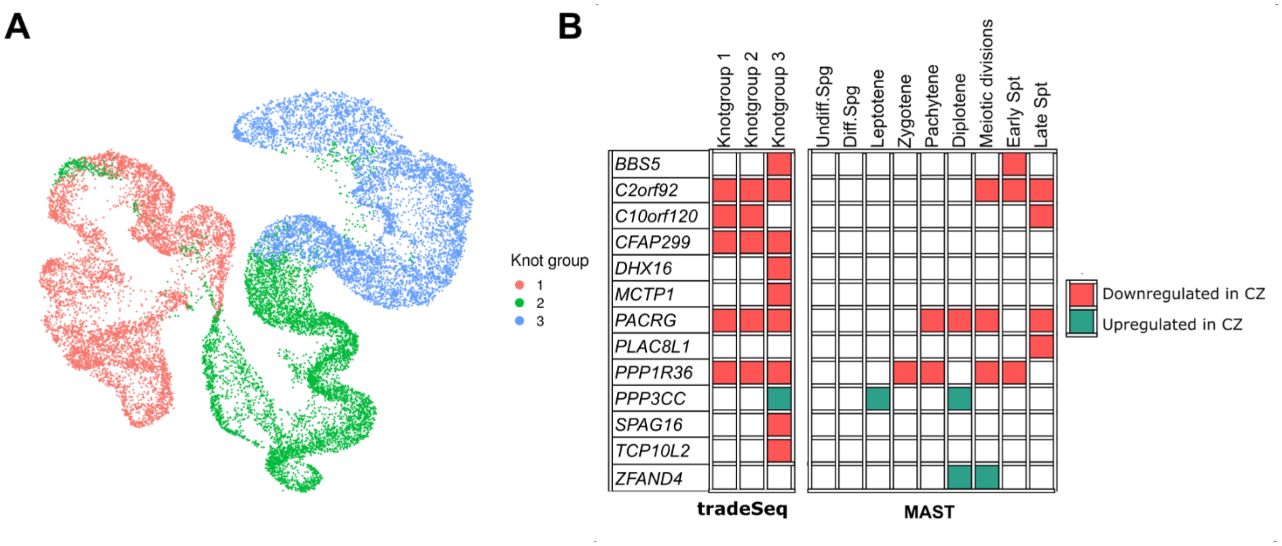

Fig. S6: scRNA seq analysis. A) UMAP plot showing the integrated CTR-CZ germ cell dataset. The cells are color-coded according to their knotgroup identity. Knotgroup 1 includes cells from undifferentiated spermatogonia to pachytene spermatocytes; Knotgroup 2 includes cells from pachytene spermatocytes to meiotic divisions; Knotgroup 3 includes cells from meiotic divisions to late spermatids. B) Schematic representation summarizing the results of the tradeSeq and MAST differential expression analyses. Red color indicates significant down-regulation of a gene, whereas green indicates significant up-regulation.

DMR27

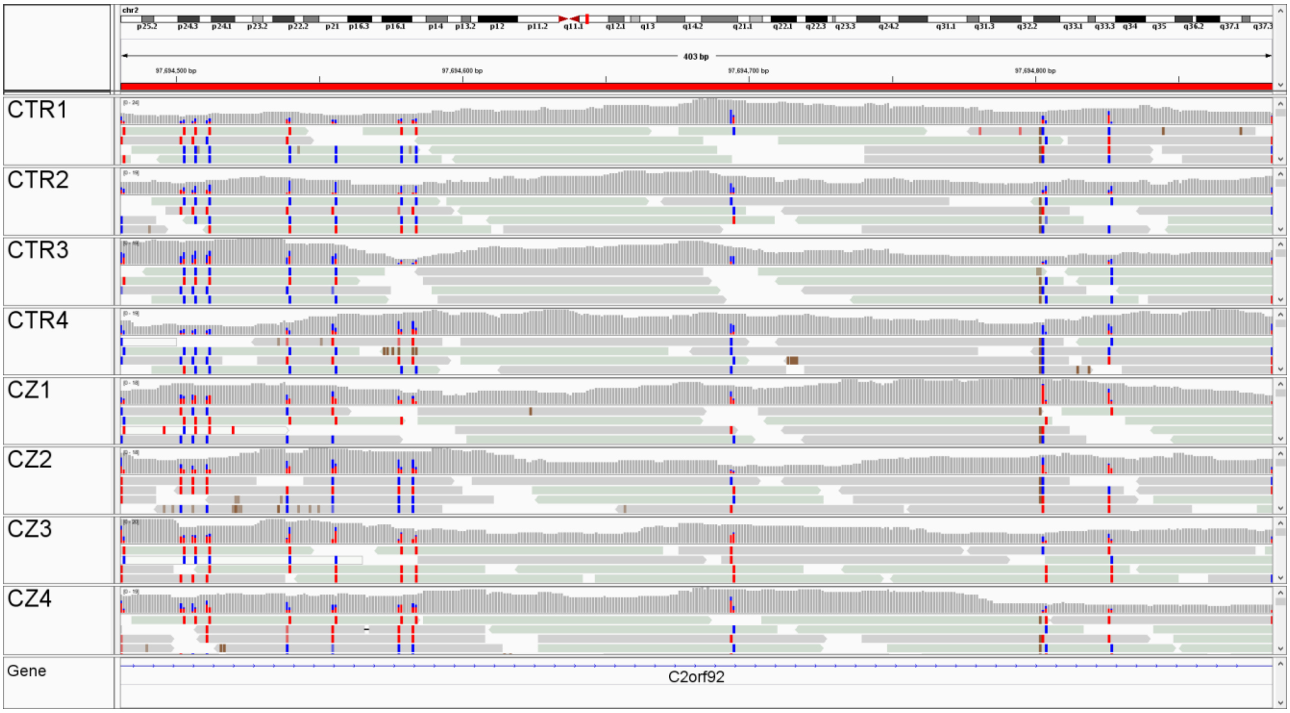

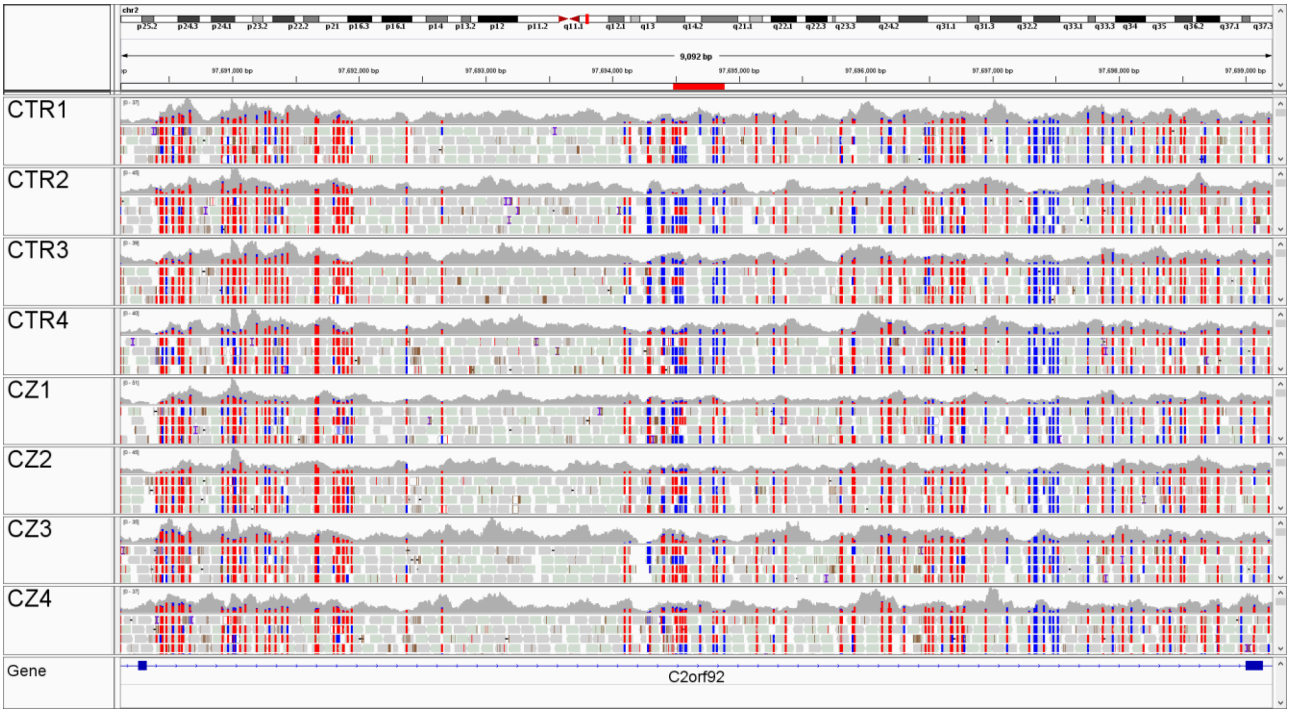

Fig. S7: (continues next page)

DMR33

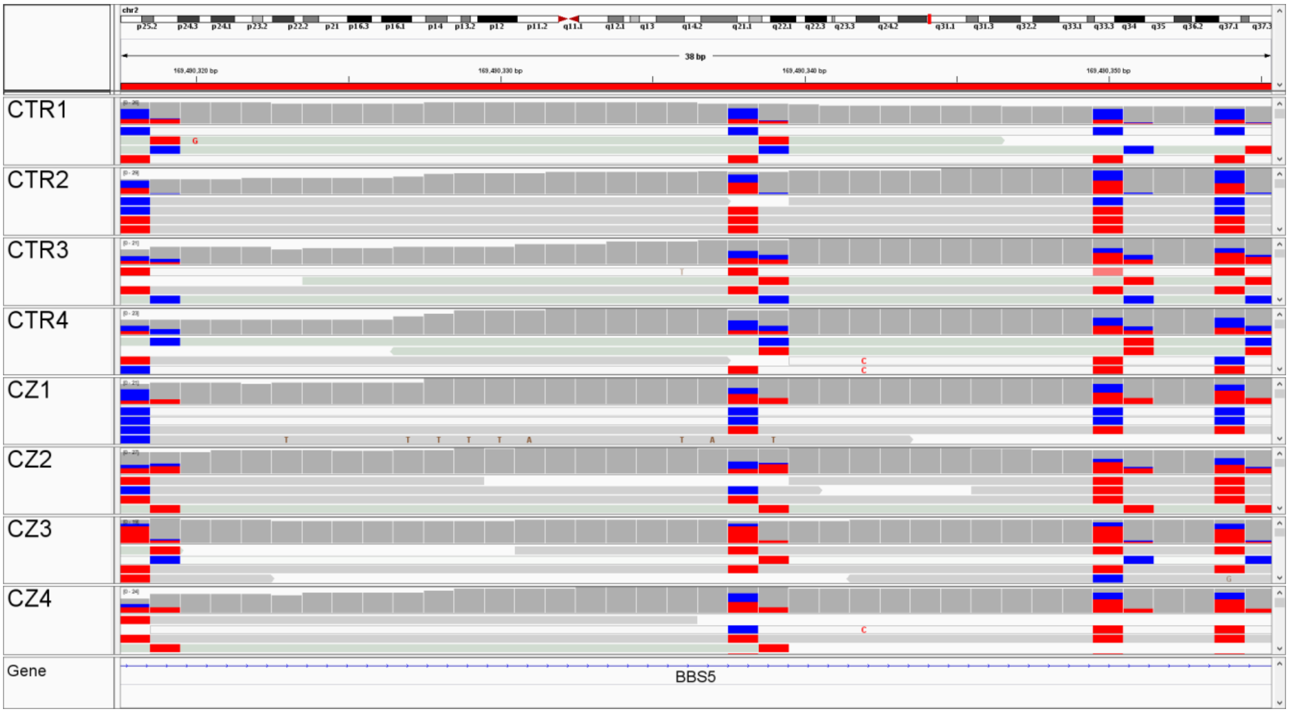

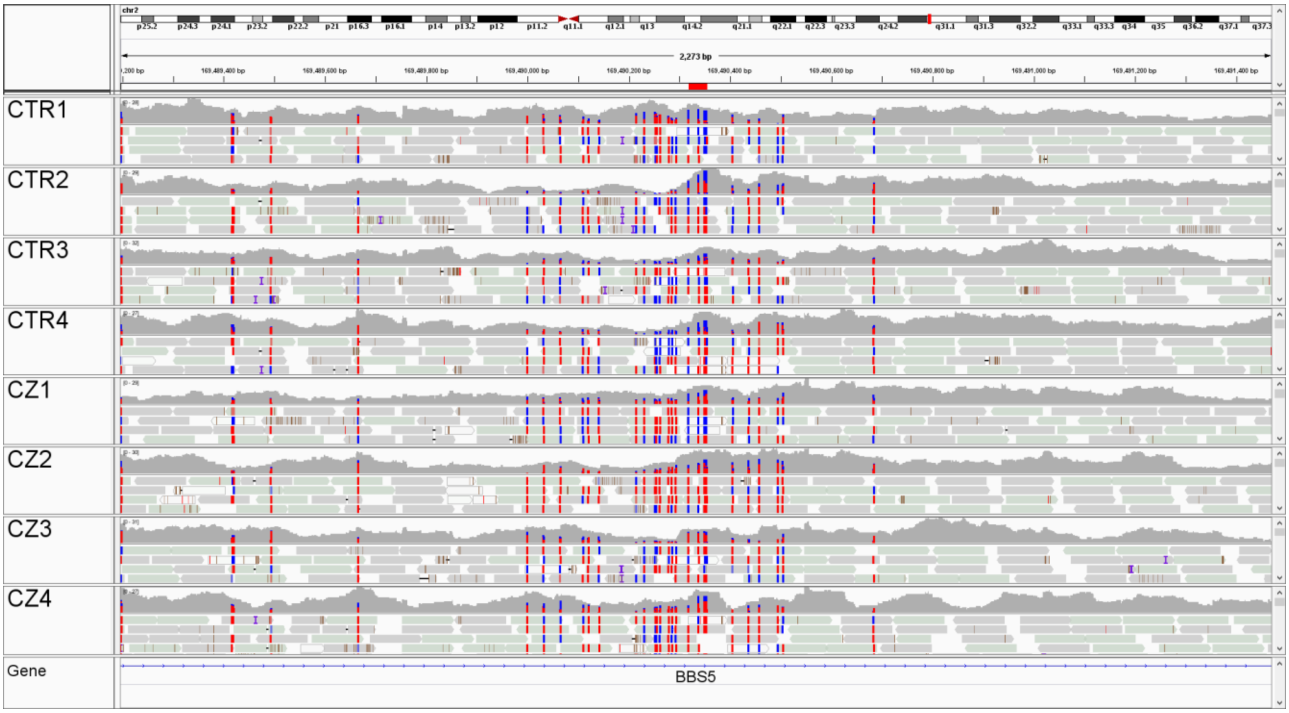

Fig. S7: (continues next page)

DMR38

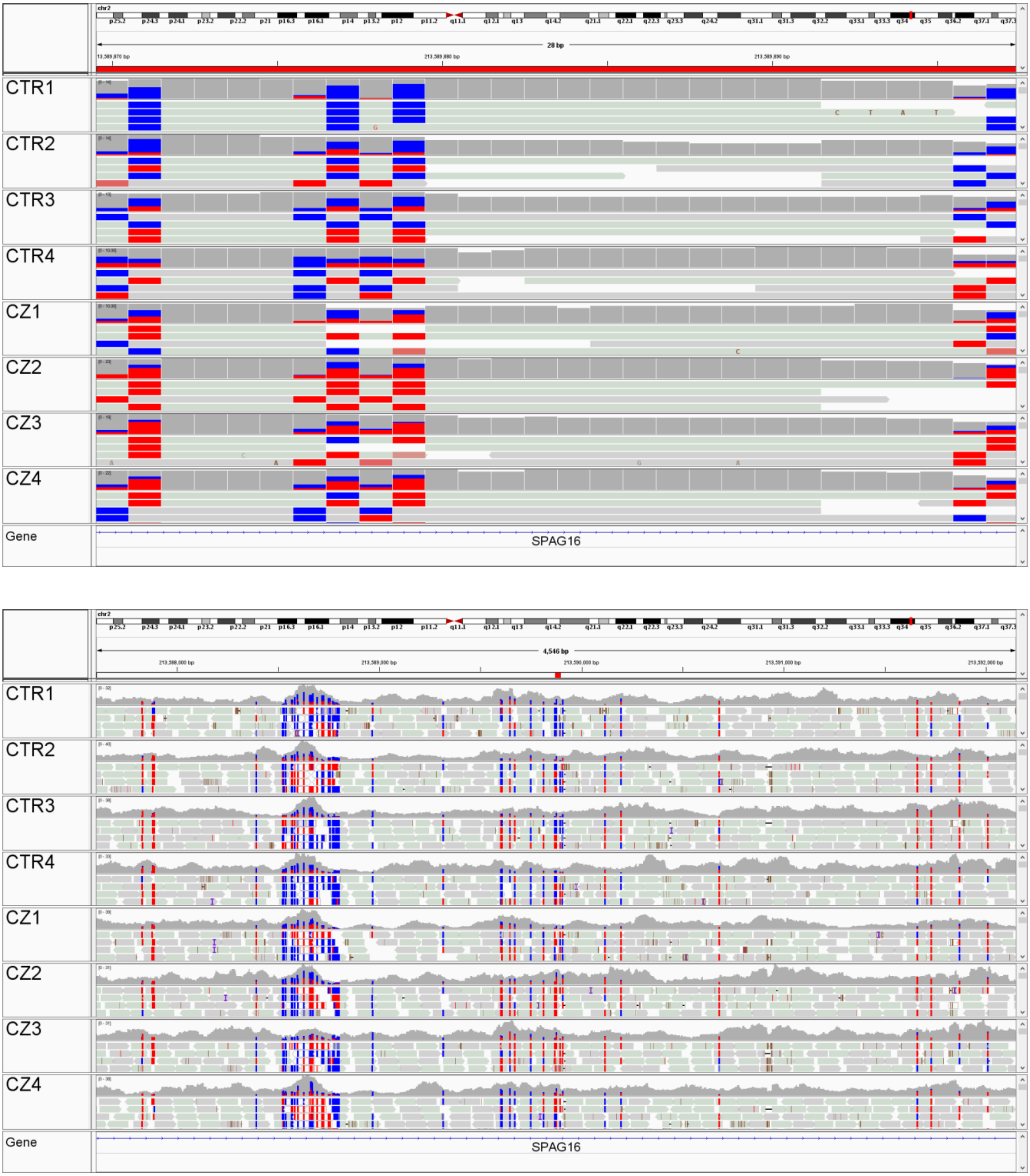

Fig. S7: (continues next page)

DMR88

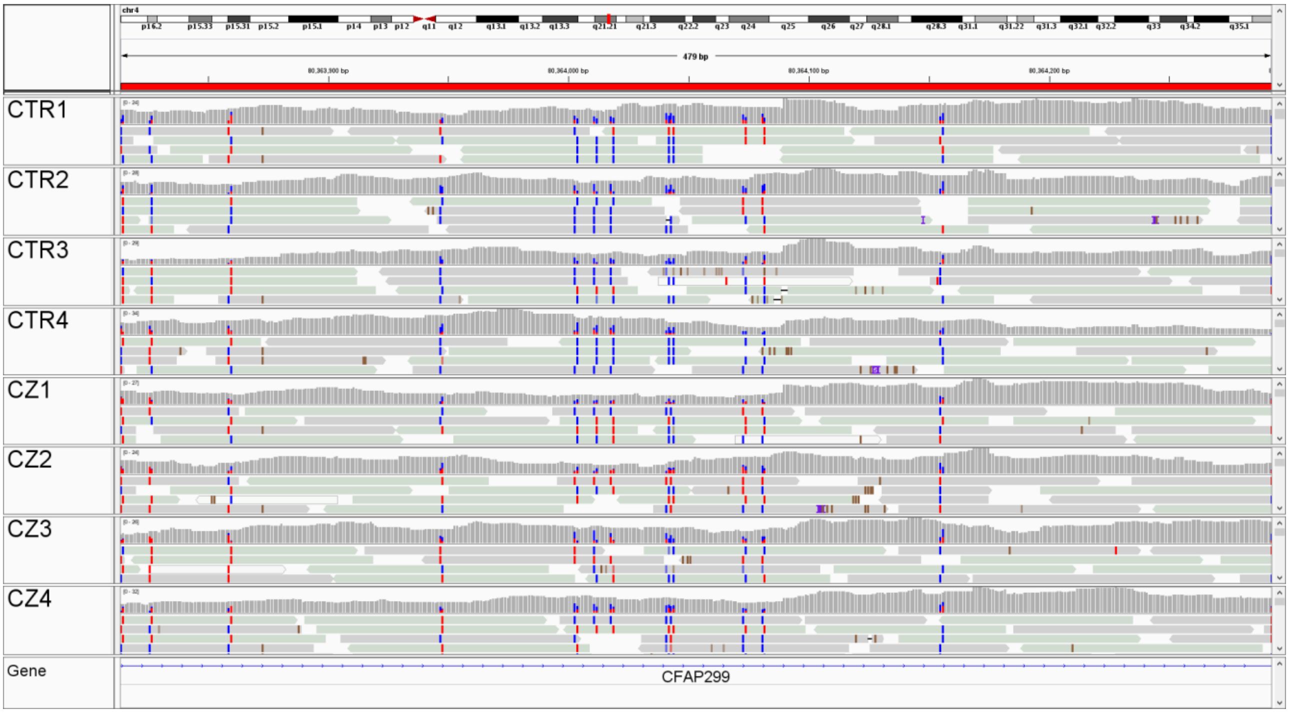

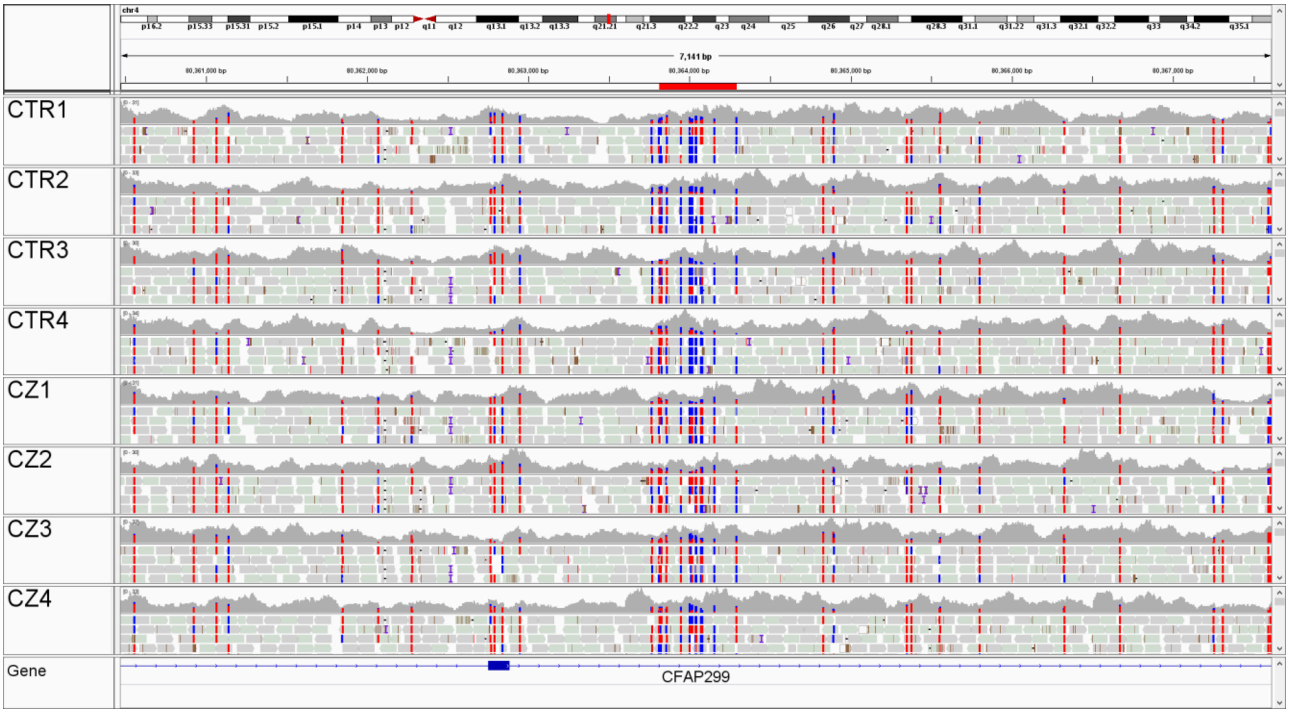

Fig. S7: (continues next page)

DMR104

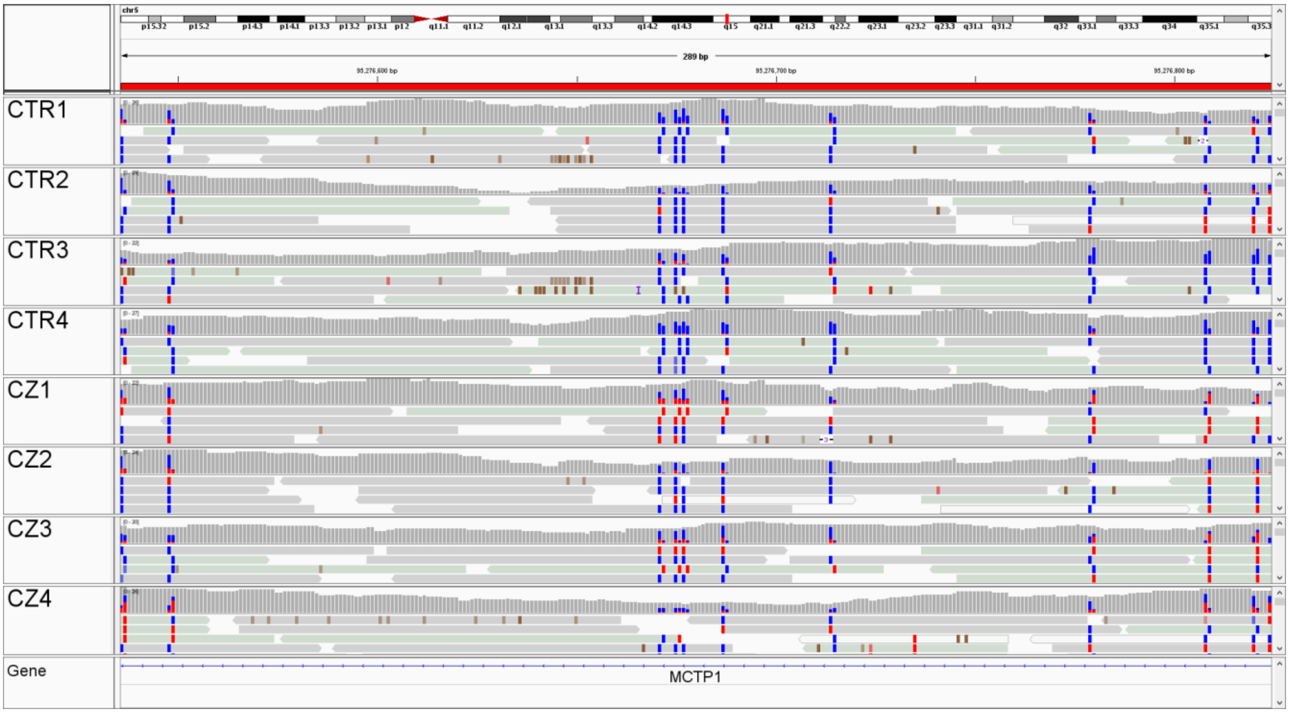

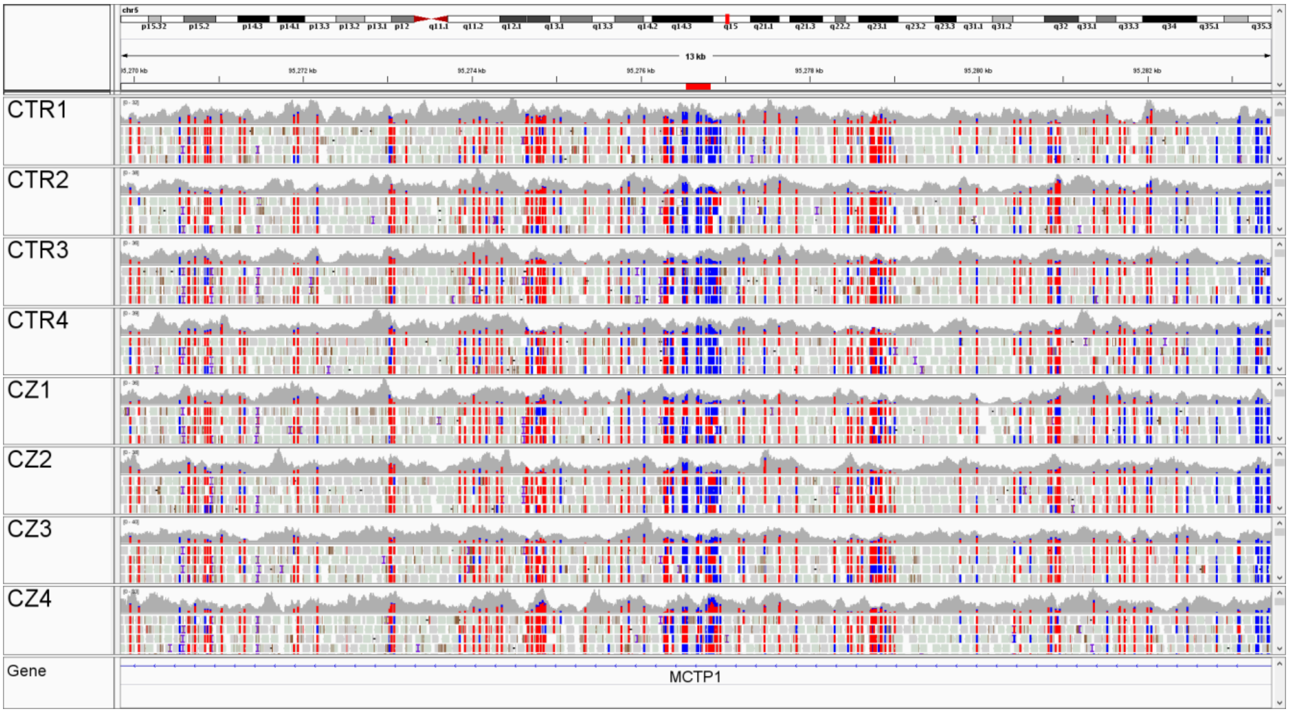

Fig. S7: (continues next page)

DMR111

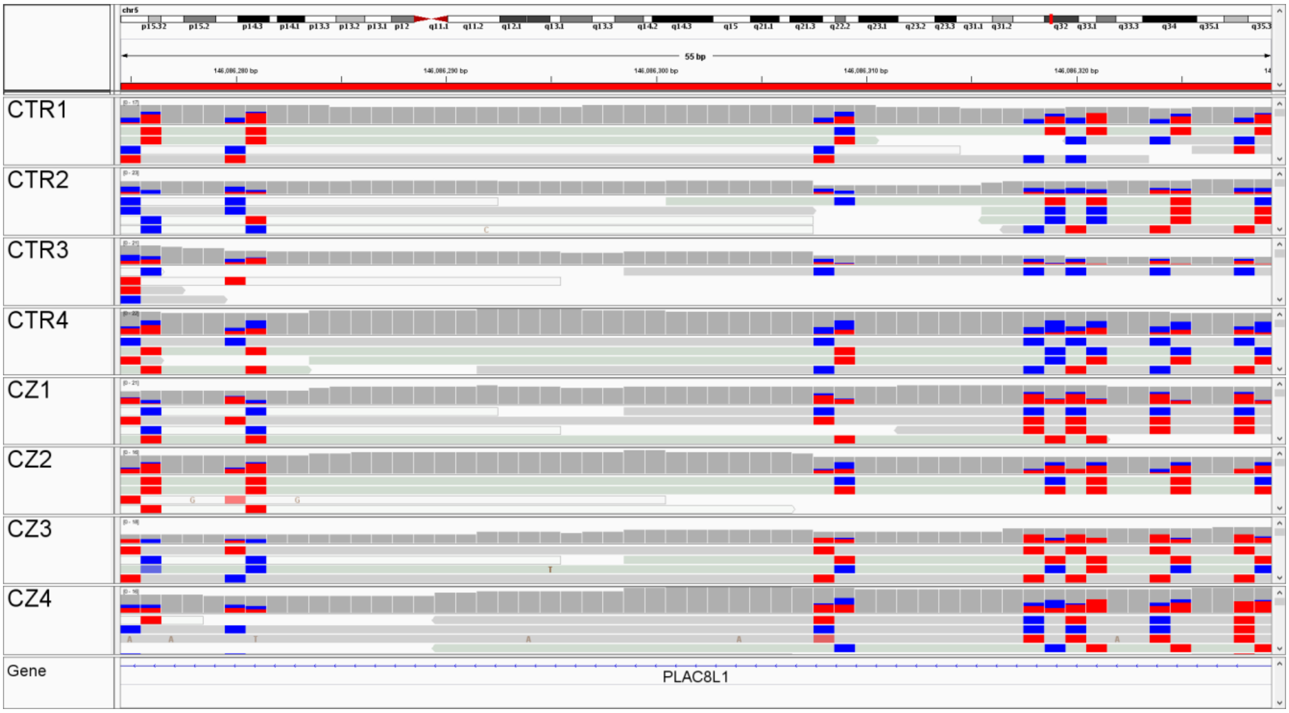

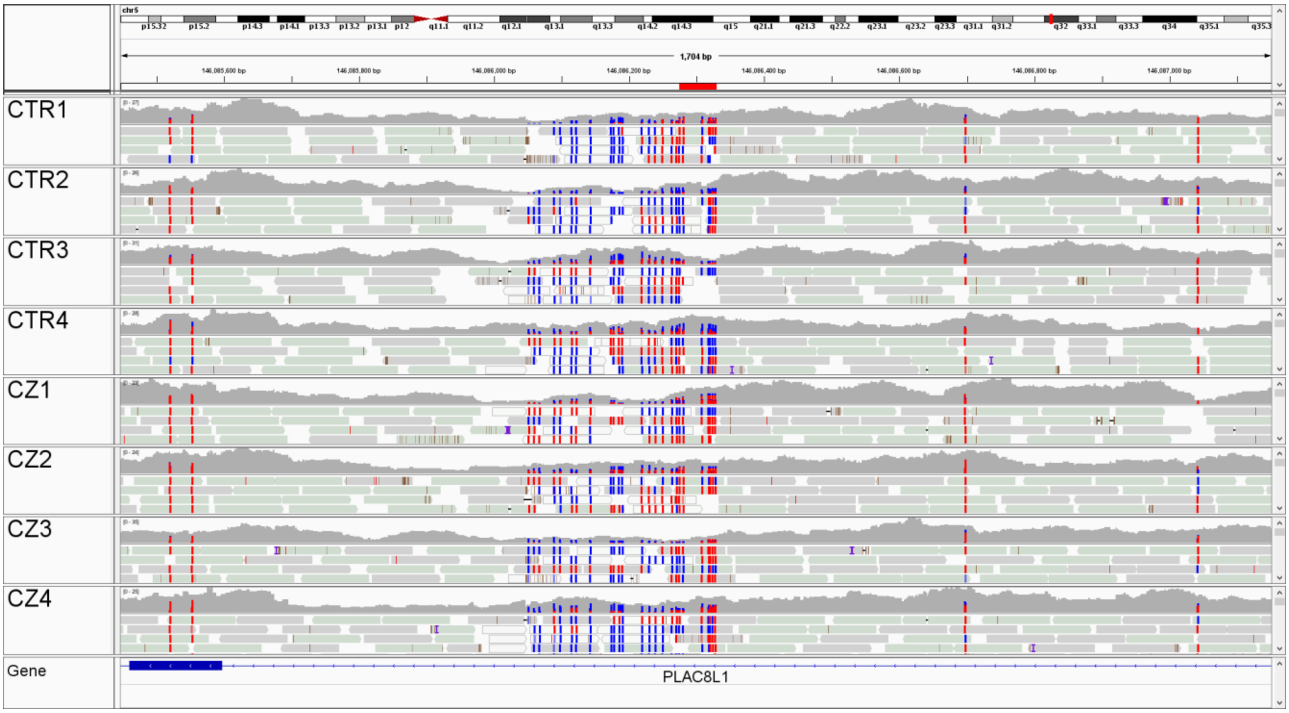

Fig. S7: (continues next page)

DMR117

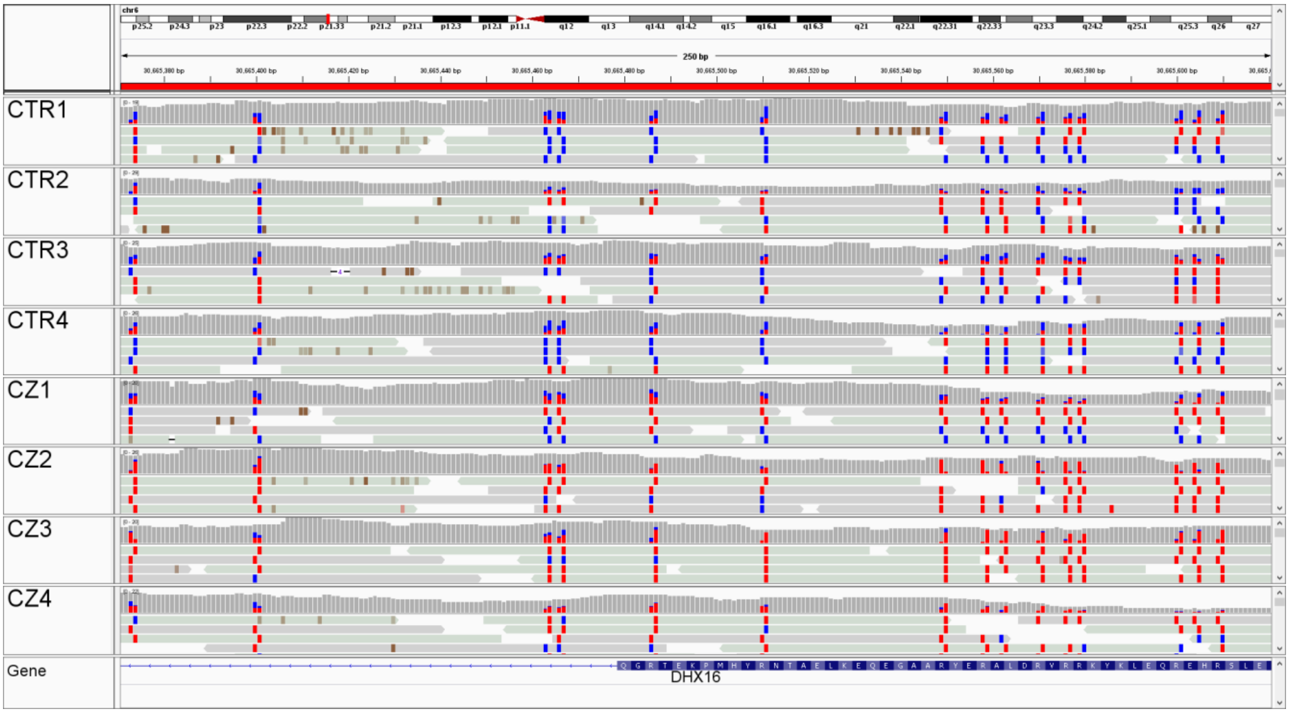

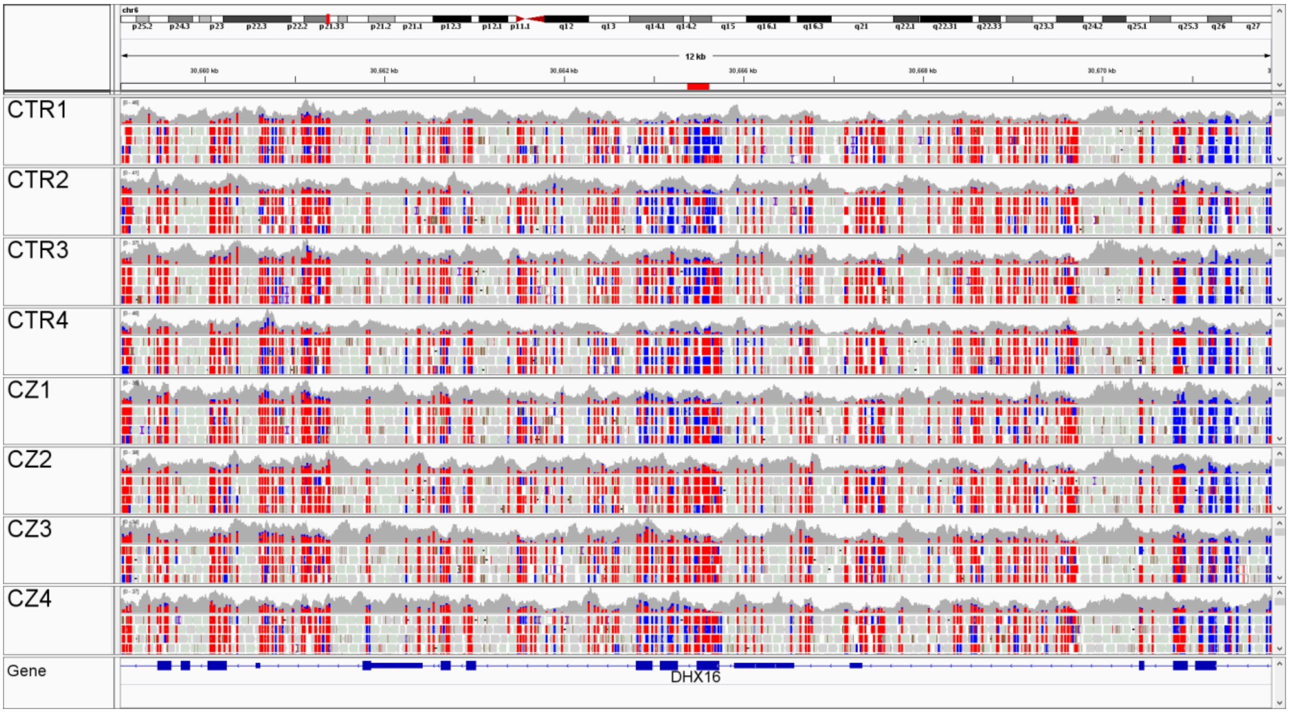

Fig. S7: (continues next page)

DMR118

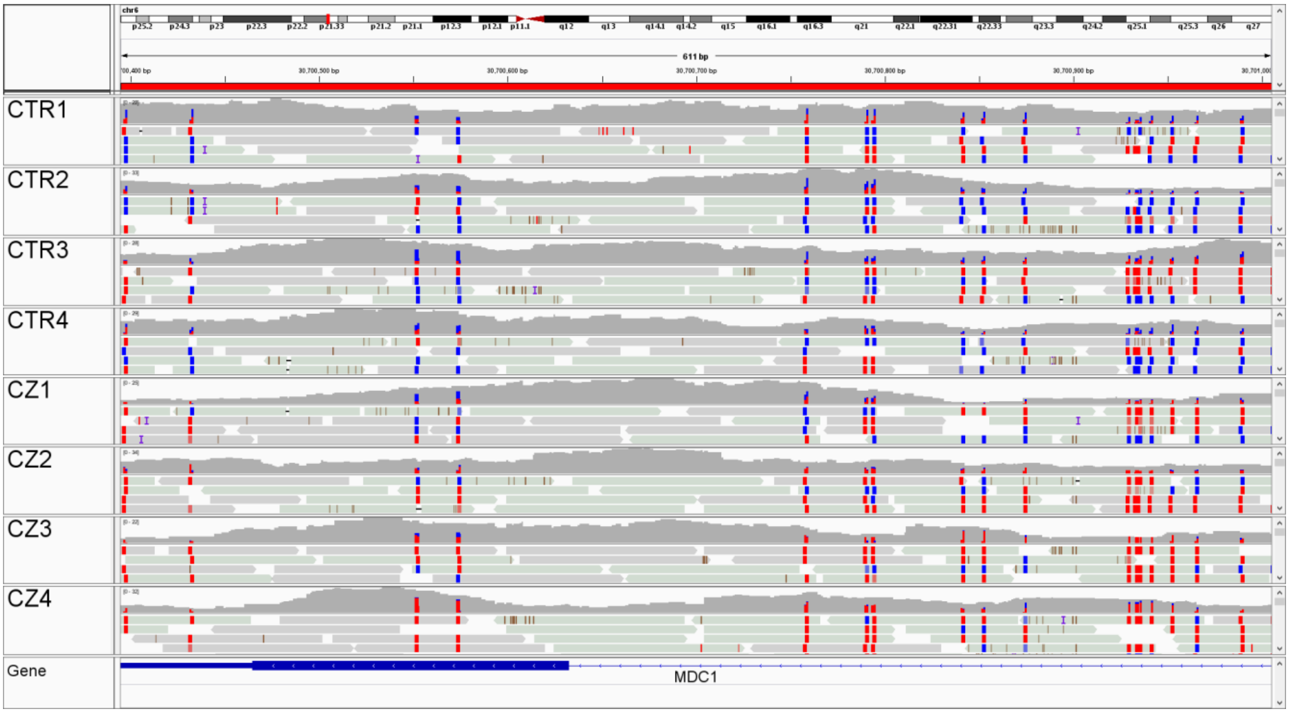

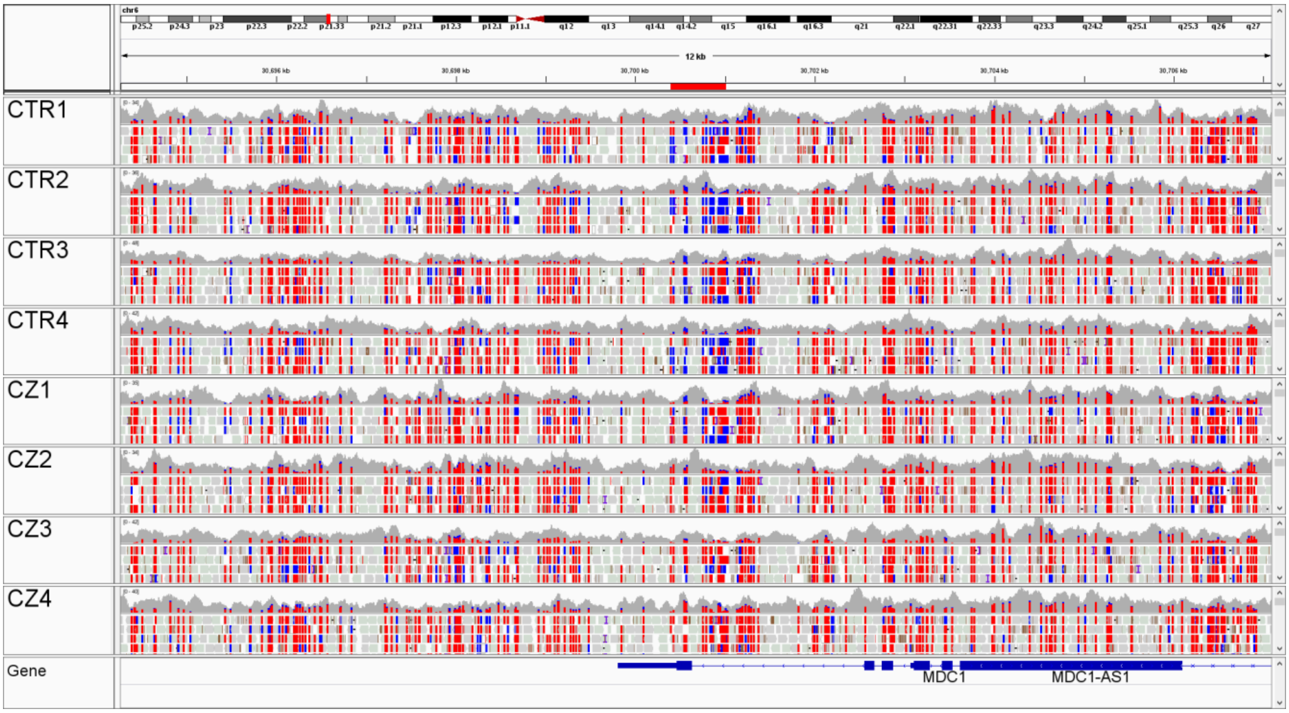

Fig. S7: (continues next page)

DMR129

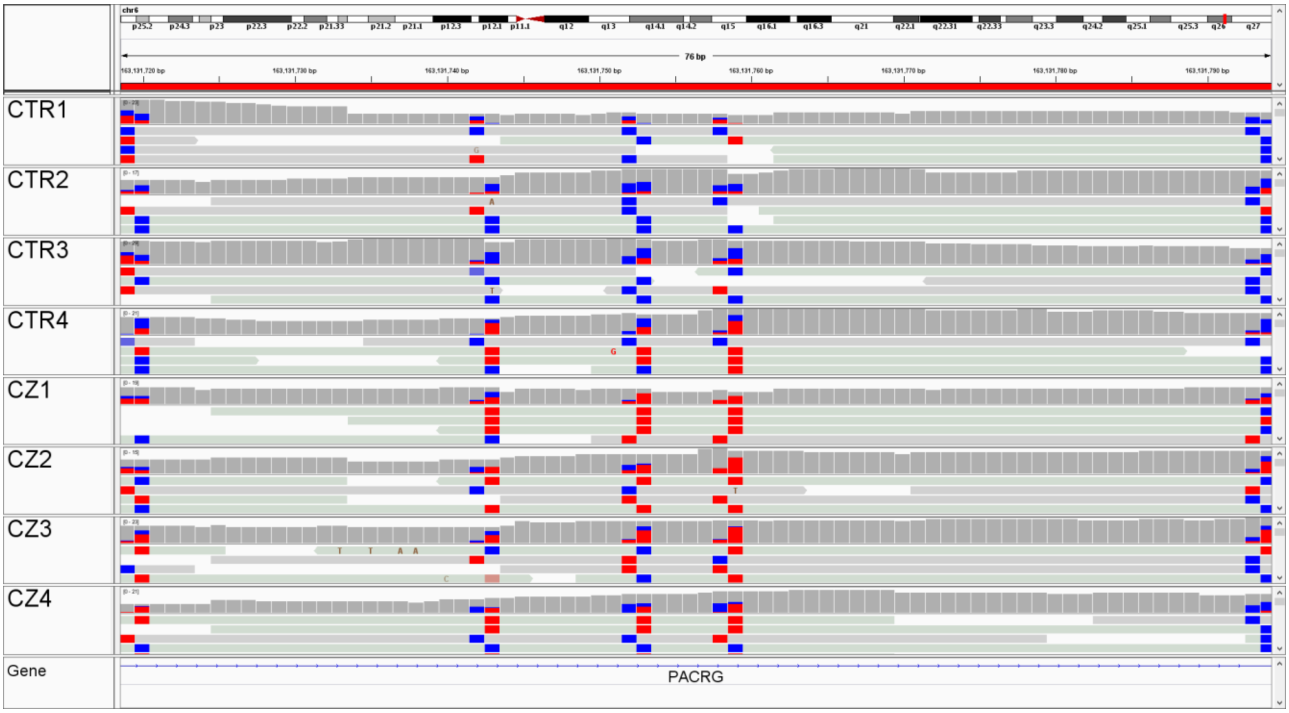

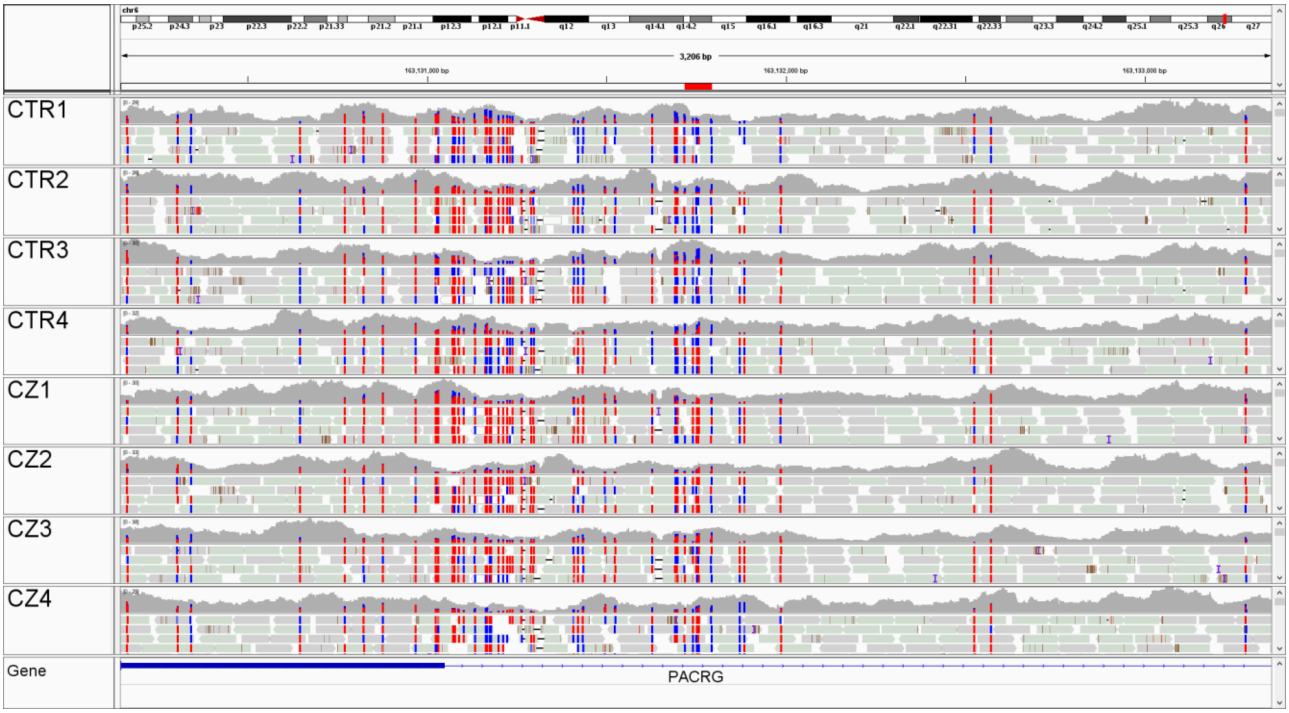

Fig. S7: (continues next page)

DMR130

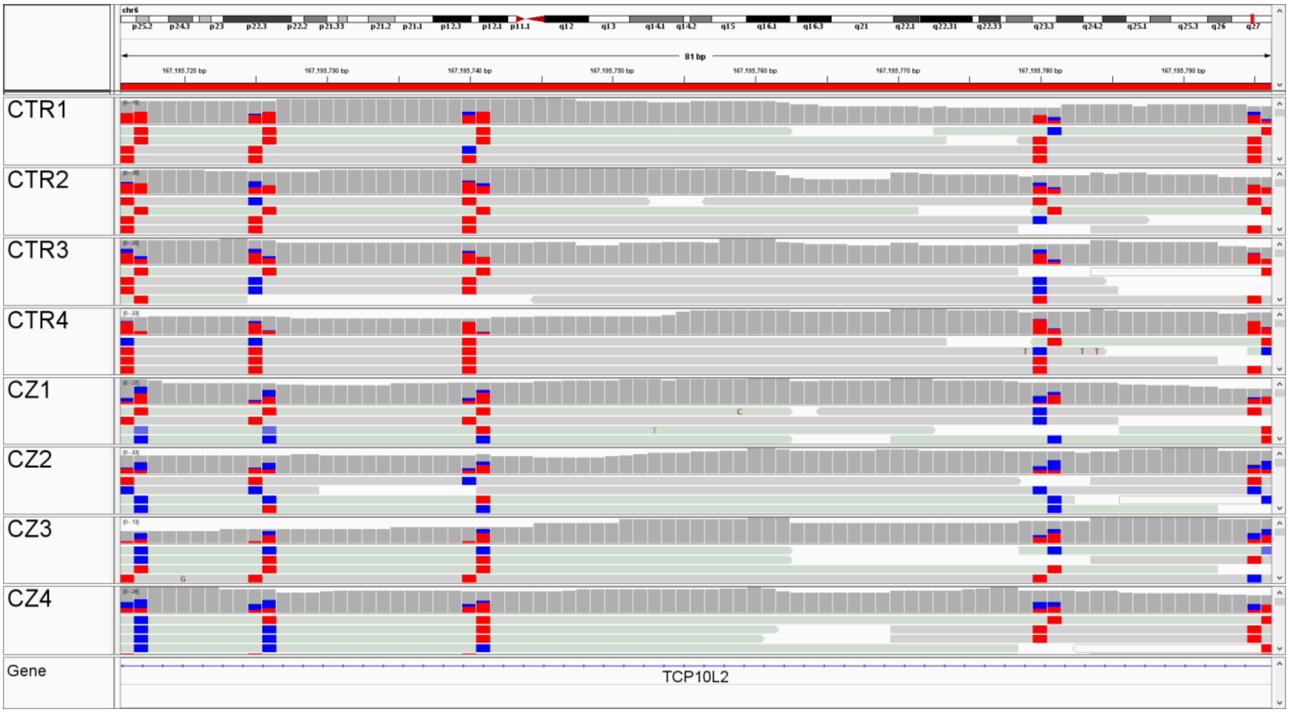

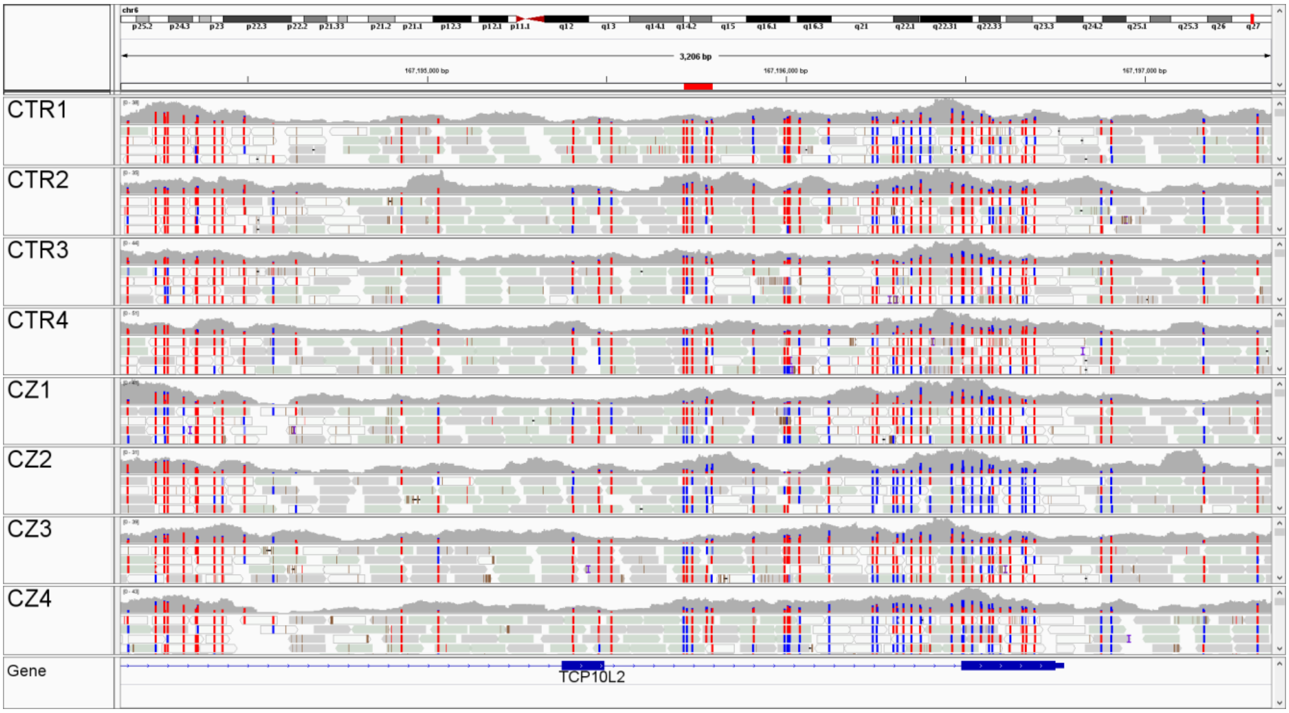

Fig. S7: (continues next page)

DMR148

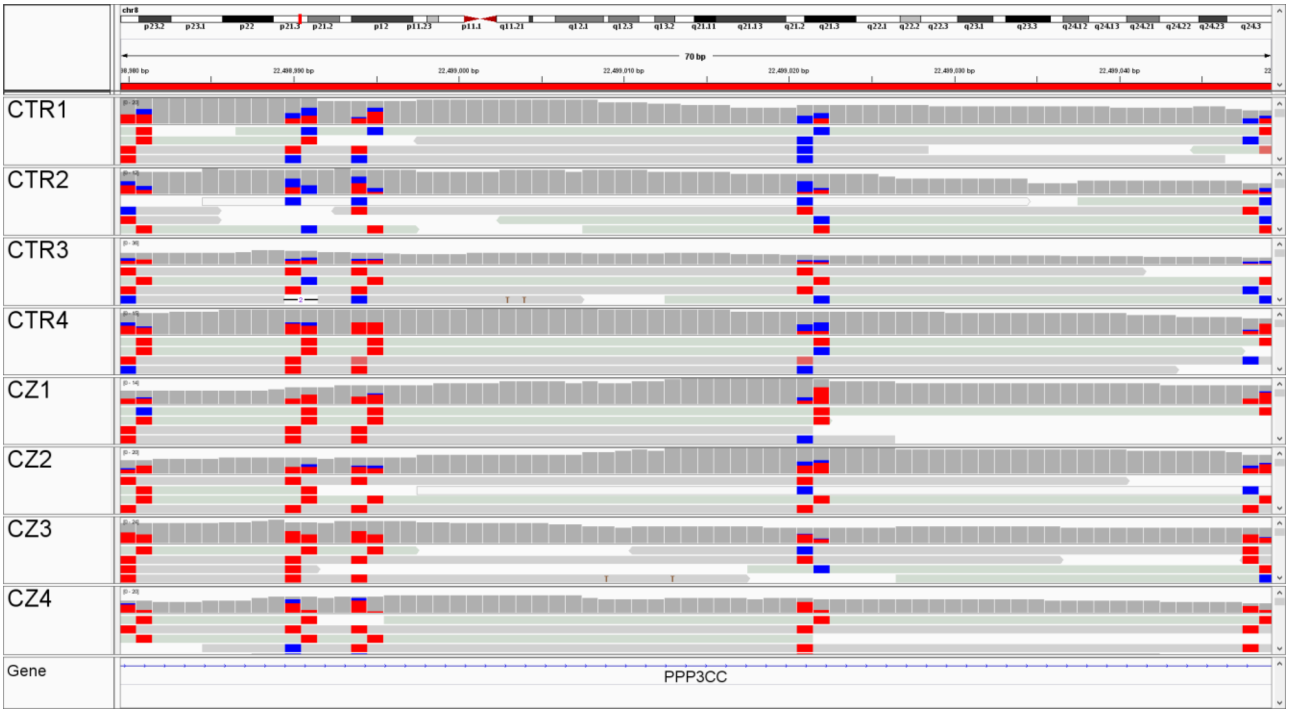

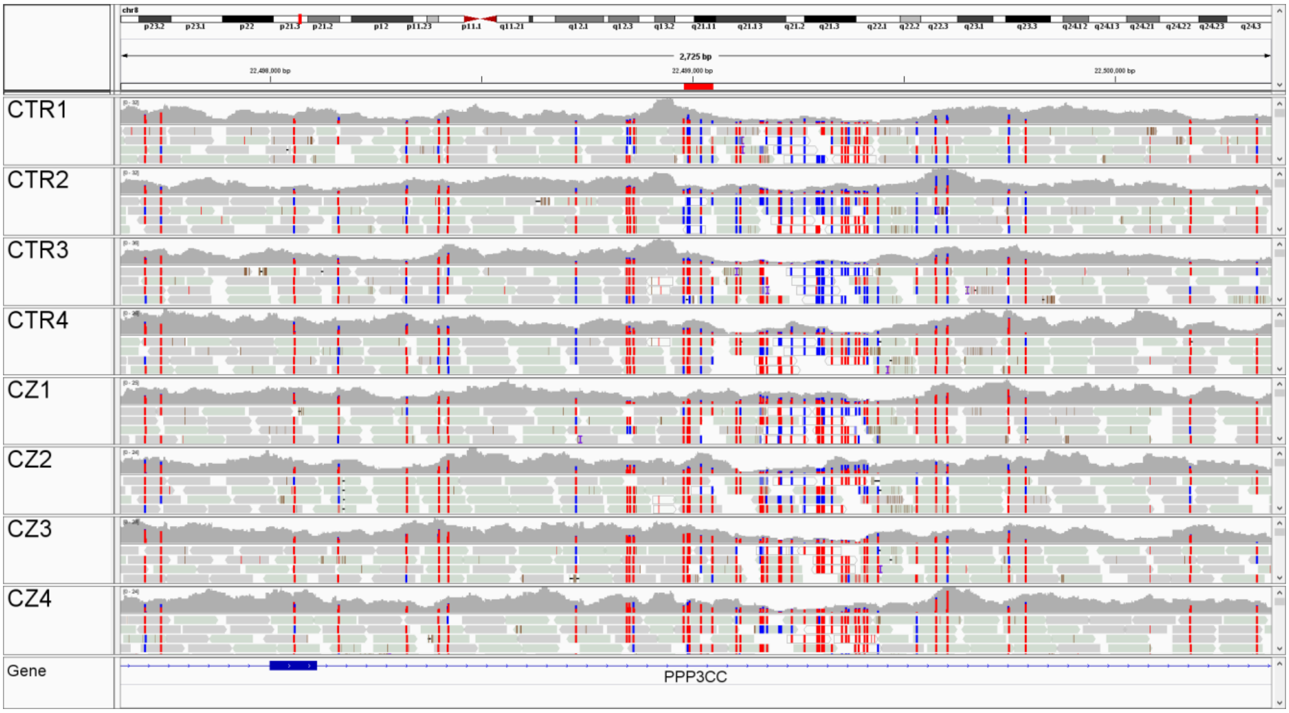

Fig. S7: (continues next page)

DMR182

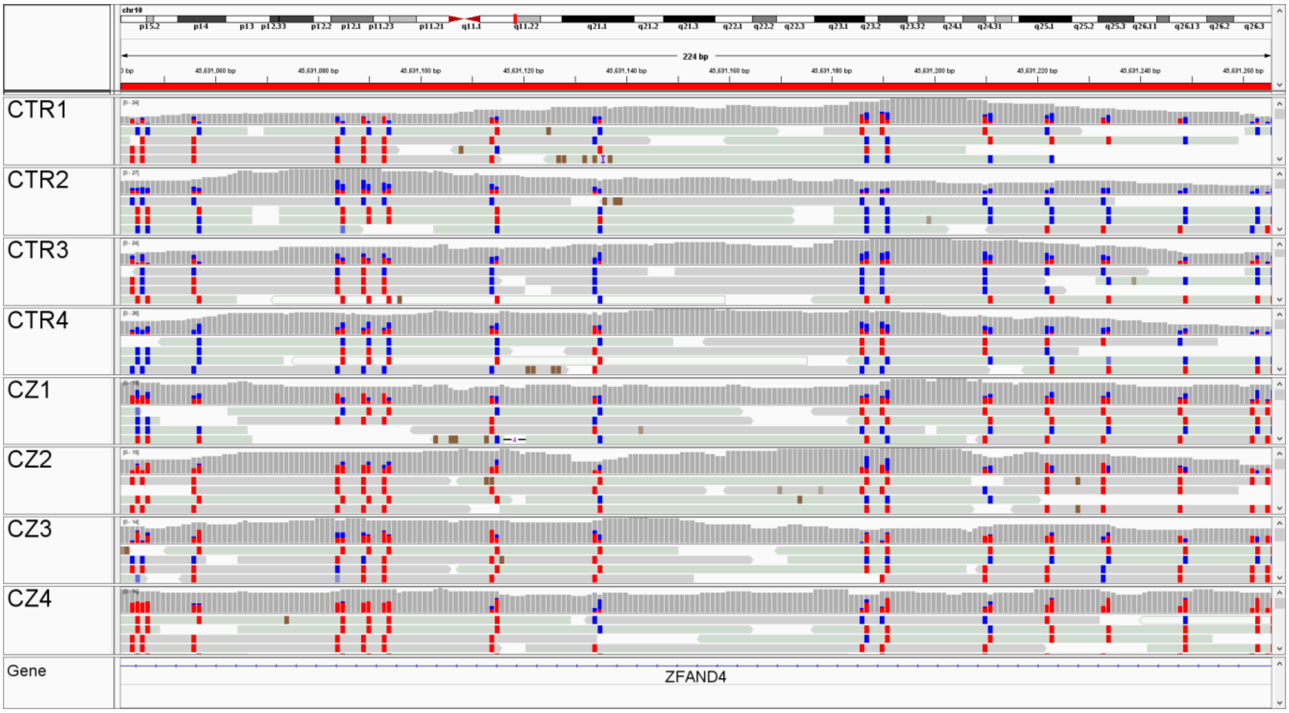

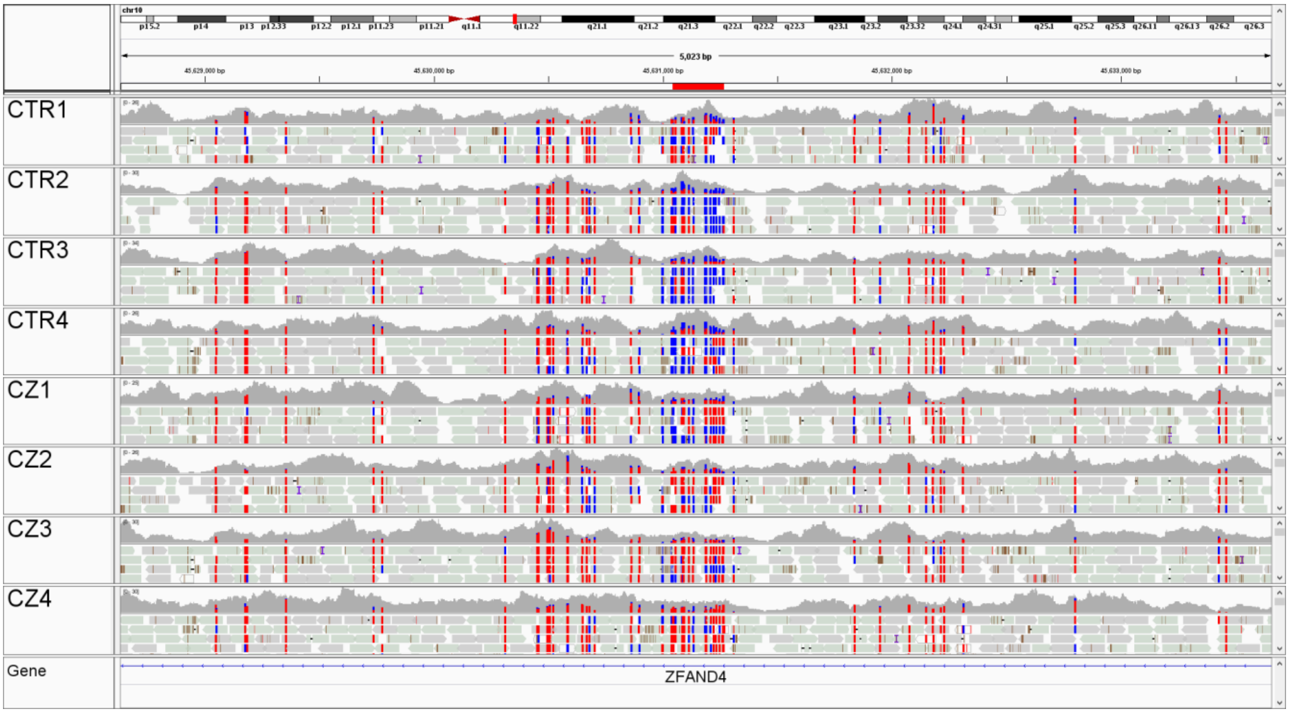

Fig. S7: (continues next page)

DMR190

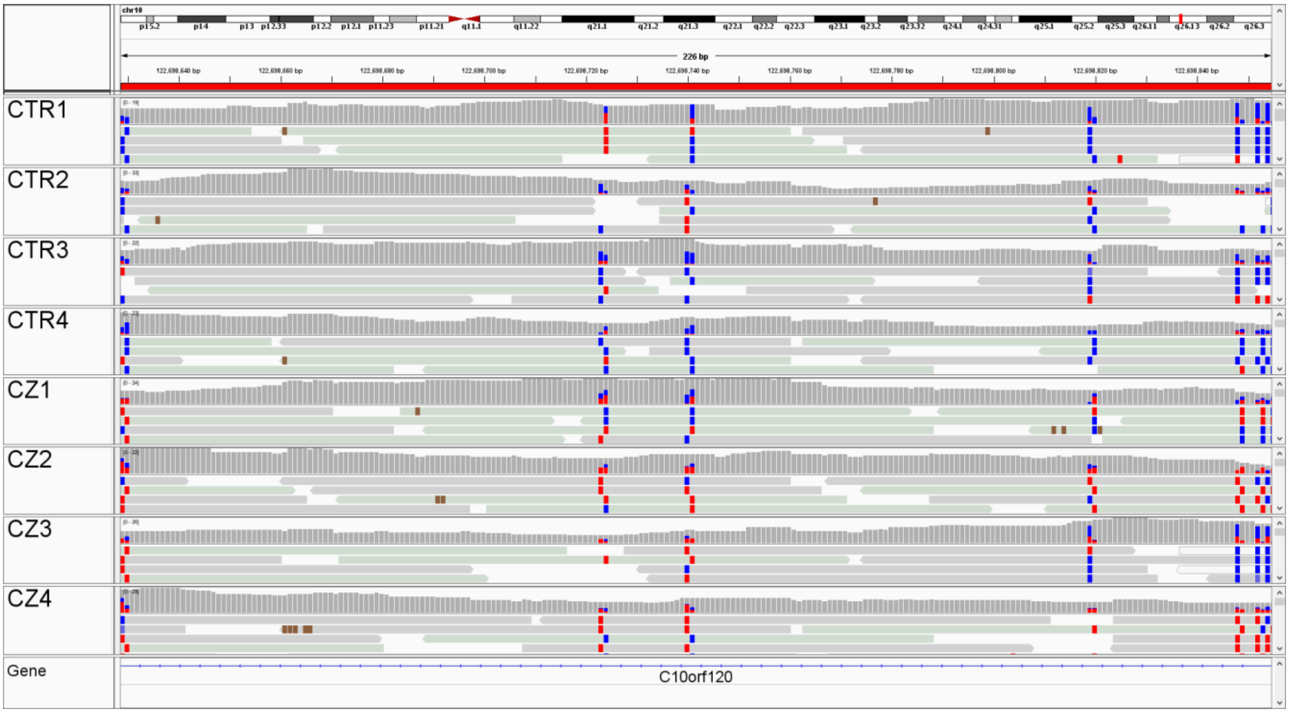

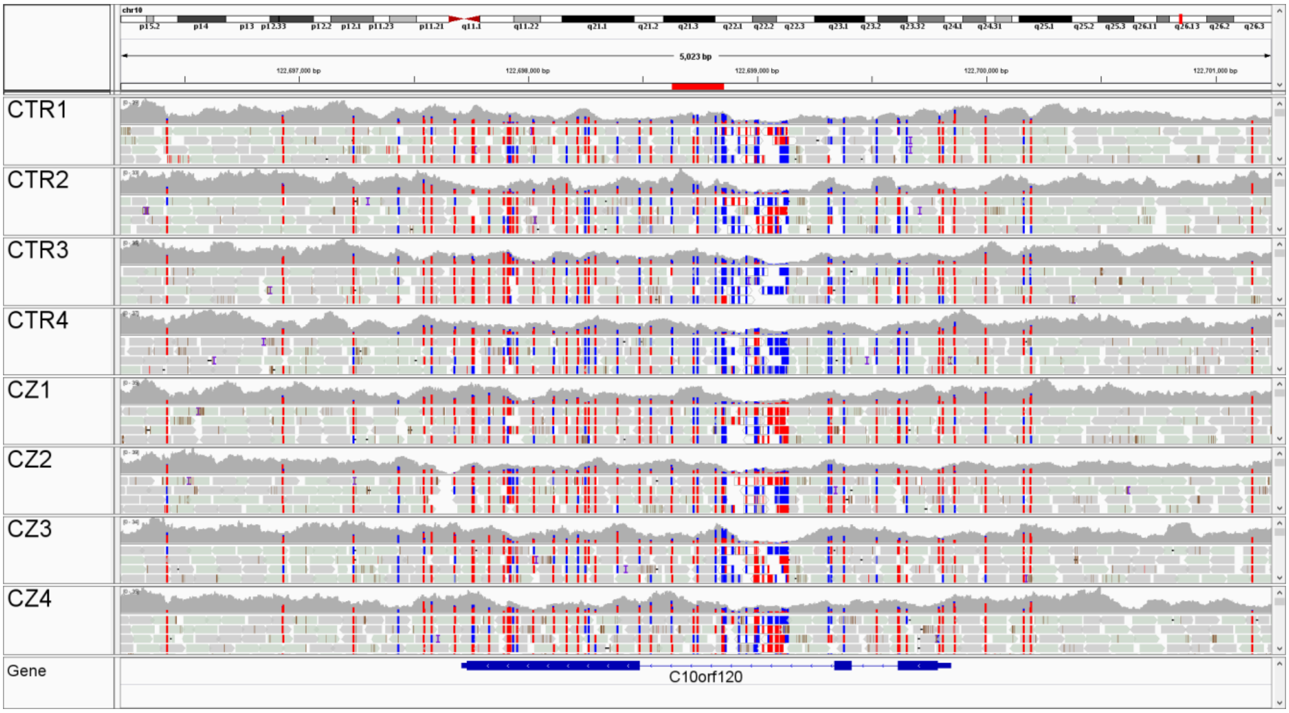

Fig. S7: (continues next page)

DMR219

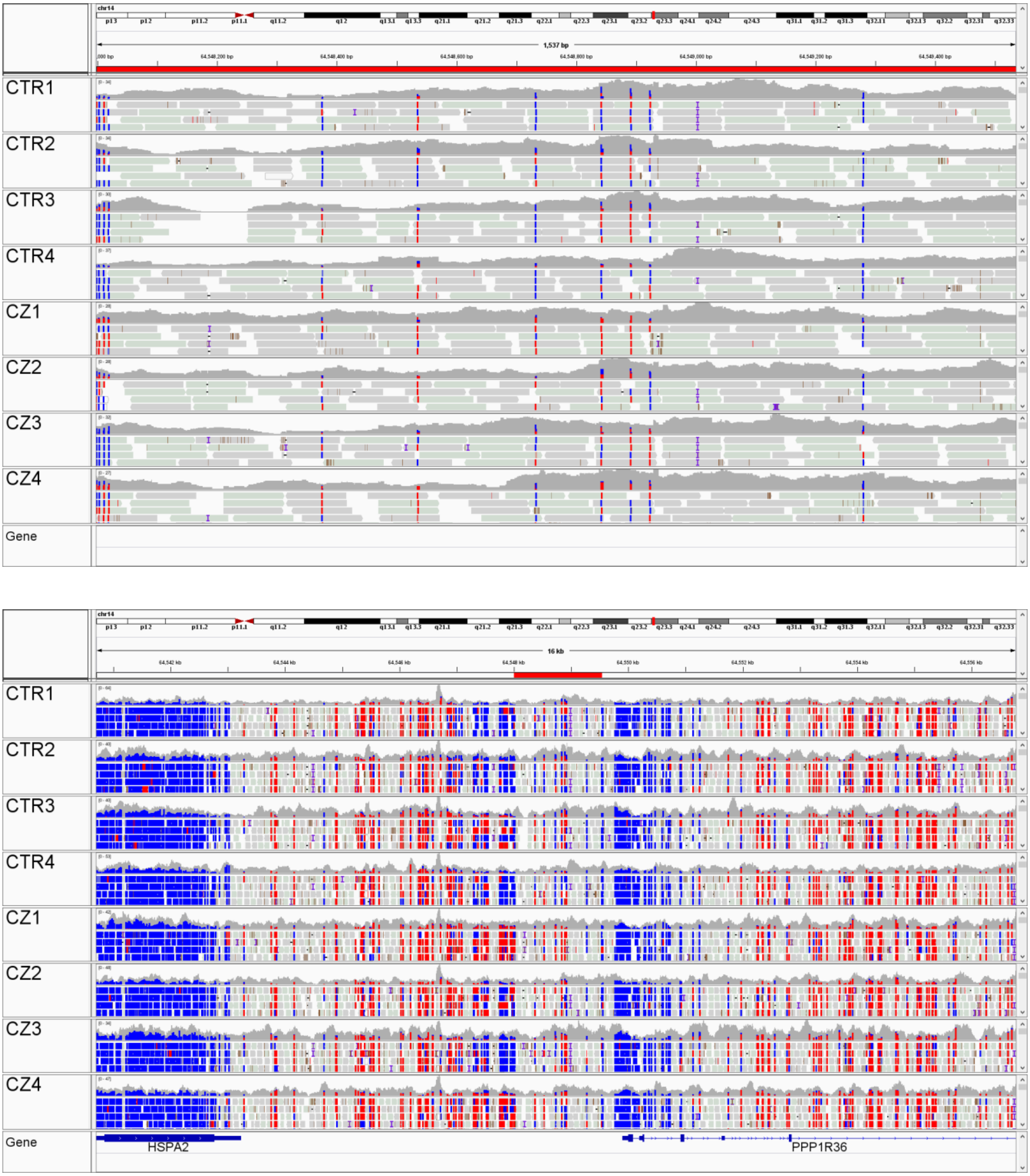

Fig. S7: IGV browser snapshots of WGBS data from control (CTR) and cryptozoospermic (CZ) testicular germ cells showing CTR-CZ DMRs associated with differentially expressed genes. Each DMR is shown as a red region either spanning the entire width (top panels) or with the surrounding genomic regions (lower panels). Only a subset of reads is shown for each sample. Methylated CpGs are shown in red and unmethylated CpGs in blue.
